# Symptom-level interactions between anxiety and internet addiction in Chinese adolescents: a large-scale network and residence difference analysis

**DOI:** 10.3389/fpsyt.2025.1558276

**Published:** 2025-09-16

**Authors:** Yi Xie, Xiao-Fei Cheng, Cong Wang, Jia Cai, Yu Wang, Yun-Fei Mu, Zhong-Yue Deng, Ai-Ping Deng, Hong-Jun Song, Xue-Hua Huang, Lan Zhang, Jun Zhang, Yi Huang, Li Yin, Wen-Wu Shen, Ming-Li Li, Mao-Sheng Ran

**Affiliations:** ^1^ Mental Health Center, West China Hospital, Sichuan University, Chengdu, Sichuan, China; ^2^ Institute of Psychiatry, West China Hospital, Sichuan University, Chengdu, Sichuan, China; ^3^ West China School of Nursing, Sichuan University, Chengdu, Sichuan, China; ^4^ West China Hospital, Sichuan University, Chengdu, Sichuan, China

**Keywords:** anxiety, internet addiction disorder, comorbidity, network analysis, adolescent, China

## Abstract

**Background:**

Anxiety and Internet addiction (IA) are prevalent and exhibit a strong correlation among adolescents. This study aimed to (a) identify key symptoms and relationships of the anxiety-IA combined network in Chinese adolescents and (b) examine the differences in anxiety-IA symptoms between rural and urban adolescents.

**Methods:**

The study was a cross-sectional survey conducted in 122 secondary schools in Sichuan Province, China, using the Generalized Anxiety Disorder Scale and the Internet Addiction Test. General network analysis and bridge network analysis were used to identify the most influential symptoms and key connectors by adopting expected influence and bridge expected influence values. Network comparison tests were conducted to explore the global strength and network structure differences between rural and urban adolescents.

**Results:**

The study included 60,268 adolescents (mean age 15.90 ± 1.65 years; 54.44% females), stratified by residence: rural (n = 49,819) and urban (n = 10,449). In the general network, “Fantasize about being on the web” (IAT15), “Neglect household chores” (IAT2), and “Reluctant to be offline” (IAT16) were the most influential symptoms. Bridge network analysis revealed that “Use the web to escape from emotion” (IAT10), “Feeling afraid” (GAD7), and “Defensive or secretive about being on the web” (IAT9) served as key connectors that bridge the comorbid network of anxiety and IA. The network structure test detected residence-related differences, and the most influential and bridging symptoms diverged by residence. “Craving for next Internet use” (IAT11) and “Irritability” (GAD6) characterized rural adolescents, whereas “Excessive worry” (GAD3) and “Restlessness” (GAD5) typified urban adolescents.

**Conclusions:**

This study offers new insights into the symptom-level interactions between anxiety and IA in adolescents. These findings also highlight the necessity of precisely addressing the comorbidity of anxiety and IA between rural and urban adolescents.

## Introduction

With the advancement of informatization, the number of Internet users in China had reached 1.092 billion by 2023, with an Internet penetration rate of 77.5% (1). The astounding development of the Internet has given rise to Internet addiction (IA), which was identified as a critical public health concern (2). IA is characterized by an excessive or poorly controlled preoccupation, urge, or behavior of Internet use resulting in functional impairments or distress (3, 4). IA can lead to physical conditions such as hearing loss, dry eye syndrome, and vision problems, while mentally it is often related to anxiety, sleep issues, and social isolation (5).

Empirical research has consistently shown that IA is often comorbid with other psychological symptoms and mental disorders, such as anxiety and Generalized Anxiety Disorder (GAD) (5, 6). Fifteen years ago, Bernardi demonstrated that of a cohort of 50 outpatients screened for IA, 15% presented with comorbid GAD (7). Adolescence marks an essential developmental stage where individuals undergo significant emotional and behavioral transformations, heightening their vulnerability to mental disorders (8). Specifically, in China, the overall prevalence of adolescents diagnosed with anxiety disorders reaches 4.7% (9). Additionally, the prevalence of Chinese adolescents screened for IA ranged from 33.37% to 45.32% (10, 11). Given the high prevalence of adolescent anxiety and IA and their positive and robust interaction (12, 13), it’s imperative to conduct in-depth research on IA and co-occurring anxiety.

A meta-analytic review indicates that social anxiety serves as a precursor to the emergence of IA among adolescents and young adults (14). Additionally, longitudinal studies have documented a consistent positive relationship between anxiety symptoms and IA over six-month intervals, with anxiety demonstrating a significant predictive capacity for IA (15, 16). On the other hand, IA has been shown to worsen anxiety symptoms (17), with studies indicating a strong link between IA severity and increased anxiety levels in adolescents (18). A variety of studies conducted among adolescents in different regions, including Peru (19), India (17), and Vietnam (20), have demonstrated a significant and positive link between the severity of IA and anxiety symptoms. Furthermore, cross-sectional studies have indicated that individuals with IA face 1.26 to 7 times the risk of anxiety compared to those without IA (21, 22). These findings emphasized the robust positive associations between anxiety and IA in adolescents, suggesting the possibility of co-occurrence of the two disorders in the population.

However, previous studies often overlooked the symptom-level interactions between anxiety and IA. Network analysis introduces a fresh approach by suggesting that disorders emerge from the interactions among symptoms, allowing us to estimate relationships across the symptoms of mental disorders (23). General network analysis focuses on characterizing the overall architecture of the symptom network by quantifying how symptoms interconnect at the whole-network level, which guides optimization and management strategies aimed at the entire system. Bridge network analysis, in contrast, is specifically concerned with identifying the critical bridge nodes and edges that knit together otherwise separable subnetworks or distinct clusters (e.g., anxiety vs. IA symptoms). Targeting these key bridging symptoms is often more efficient, which can simultaneously dampen risk across multiple subnetworks, thereby preventing the spread of pathology. Thus, the two approaches are complementary and mutually indispensable.

In these network models, symptoms are represented as nodes linked by edges, where these edges reflect the connections between symptoms, considering the influence of all other nodes in the network (24). The central nodes within this network identify important symptoms that are essential to the underlying mechanisms of the disorder’s pathology (25). Moreover, symptoms that connect both disorders—known as bridge symptoms—determine comorbidity and offer prime targets for clinical interventions (26).

Previous research has shown that symptoms like “Academic decline due to Internet use” and “Feeling depressed, moody, or nervous only while offline” are essential to the underlying mechanisms of anxiety combined with IA network pathology in nursing students (27). Besides, an investigation involving children and adolescents through school recruitment identified “Feeling afraid” as a key connector that bridged the IA and anxiety combined network, suggesting that addressing fearfulness can disrupt the spread or exacerbation of symptoms across anxiety and IA (12). It’s observed that the symptoms most requiring intervention in the IA and anxiety comorbidity network vary across populations. Therefore, further investigation is warranted within a large, representative sample of Chinese adolescents to clarify which specific symptoms most critically demand intervention in the comorbid anxiety-IA.

Furthermore, recent research has also underscored residence-specific disparities in anxiety and IA. Among middle-school students in China, both the prevalence of social anxiety and IA are significantly higher in urban adolescents than in their rural counterparts (28). Among 692 Polish respondents (mean age = 20.8 years), those at risk for IA who lived in rural areas exhibited significantly more severe psychopathological symptoms, including phobic anxiety, than their urban counterparts (29). While analyses stratified by residence revealed no significant differences in the combined anxiety-IA network structure among nursing students (27). However, the symptom-level nuances of comorbid anxiety-IA networks across residential settings remain insufficiently explored. These mixed findings underscore the need to examine how specific symptom pathways differ between rural and urban adolescents, meriting targeted investigation in large, representative samples of adolescents.

Thus, in the present study, we used general and bridge network analysis to (a) identify the most influential symptoms and connectors in the anxiety and IA combined network and (b) compare the structure of anxiety-IA networks between rural and urban adolescent students in China. It could provide the most critical, precise targets for clinical intervention in adolescent students.

## Methods

### Research design and participants

This convenience-sampled cross-sectional study was conducted among adolescent students in Sichuan Province, China, from December 14, 2022, to February 28, 2023. A total of 122 middle schools and high schools participated in the survey, ensuring a broad and diverse sample that spans 20 of the 21 prefecture-level divisions in Sichuan Province, with a 95% coverage rate; only the Ganzi Tibetan Autonomous Prefecture was not represented. The criteria for inclusion included (1) middle and high school students in Sichuan Province, China, (2) being able to read and fill out the questionnaire, and (3) voluntarily participating in the study. The exclusion criteria included (1) not being enrolled in schools in Sichuan Province, (2) having difficulty filling out the questionnaire, and (3) being unwilling to take part in the study.

An online survey was distributed through SoJump.com (wjx.cn), a viable source of convenience samples in China (30). To enhance the quality of the data collection, the survey information was initially sent to teachers at the participating schools. These teachers then scheduled their classes into the schools’ computer rooms orderly, where students completed the survey under teacher supervision (31–33). A total of 65,509 students accessed the survey, and 60,268 (92.0%) completed the questionnaires after providing informed consent, while 5,241 (8.0%) declined to participate or did not complete the survey.

Informed consent was obtained from all participants themselves, their parents, and their teachers, who served as guardians in school, and the participants’ anonymity has been preserved. The study received ethical approval from the Research Ethics Committee of West China Hospital, Sichuan University (No. 2022 – 1790). All procedures conformed to the provisions of the Declaration of Helsinki (as revised in Brazil in 2013).

### Measurements

#### Anxiety

In assessing anxiety, this study utilized the 7-item Generalized Anxiety Disorder Scale (GAD - 7), which rates symptoms on a scale from 0 (none) to 3 (almost every day), reflecting the frequency of anxiety symptoms experienced in the past two weeks (34). The total score ranges from 0 to 21, with higher scores denoting more severe anxiety (35). The GAD - 7 scale has demonstrated strong reliability among Chinese youth, with a Cronbach’s α range of 0.71 to 0.87 (36) and a higher Cronbach’s α range of 0.97 in our sample.

#### IA

This research applied Kimberly S. Young’s 20-item Internet Addiction Test (IAT - 20) for assessing IA, which was developed based on the DSM-IV-TR criteria for pathological gambling (4). Scored on a 5-point Likert scale, from “very rarely” to “very frequently,” the IAT - 20 yields a total score between 20 and 100 (4), with a higher score indicating a higher level of IA. The IAT - 20’s validity and reliability were confirmed among Chinese adolescents, with a *Cronbach’s α* reaching 0.93 (37) and a slightly higher *Cronbach’s α* reaching 0.96 in our study.

Given the controversial factor structure and potential issues within the original items, Khazaal’s modifications to the IAT - 20 were used for data analysis (38). Specifically, the modifications involved combining items 3 and 19 and items 6 and 8 and renaming them IAT3 and IAT6, respectively. For the combined items, the highest or only responses were used. Since item 4 focused on making friends online, it was considered outdated and deleted. In addition to these five items, other items from 1 to 20 were retained with their original names, resulting in a modified 17-item Internet Addiction Test scale (IAT - 17) (38). The IAT - 17 scale exhibited high reliability in our study, with a *Cronbach’s α* of 0.95.

#### Other features

We used a self-designed questionnaire to collect self-reported data like demographics (age, sex, grade, and ethnicity), academic satisfaction, and family background (single child, family monthly income, marital status of parents, and parenting style).

### Statistical analysis

Descriptive and network analyses were carried out using R programming, version 4.5.1. Categorical variables were analyzed with chi-square tests and Mann-Whitney U tests, and continuous variables were assessed using independent-sample t-tests. A threshold of.05 was defined as significance for all two-tailed tests. Our main approaches included setting up general and bridge network models, assessing the accuracy and stability of our models, analyzing the most influential symptoms and connectors, and comparing the differences between the rural and urban areas.

#### General network model

As for general network analysis, we utilized the “glasso” package for modal estimation (39), with the “qgraph” package for the model visualization (40). The “EBICglasso” algorithm combines the Extended Bayesian Information Criterion (EBIC) with the graphical Least Absolute Shrinkage and Selection Operator (gLASSO). EBIC selects the penalty value that yields the most parsimonious yet well-fitting model, thereby minimizing false-positive edges and enhancing interpretability, and gLASSO introduces a penalty parameter that shrinks small partial-correlation estimates (0.001 in our study) to exactly zero, producing a sparse network (41).

When each node (a node represents a symptom) is connected to several other nodes through edges (an edge represents specific links between two symptoms) with different weights, the final network is constructed automatically and indicates the centrality indices of each node. We provided quantitative centrality indicators for each node based on the unique configuration of a network, including Expected Influence (EI), Strength, Betweenness, and Closeness. EI is the sum of the signed edge weights (EW) connected to a node, capturing both the magnitude and the valence (positive or negative) of its associations with all other nodes. Strength is the sum of the weight of all direct connections between a specific symptom and others. Betweenness indicates how often a symptom lies on the shortest indirect path to another node. Closeness indicates how strongly a node is indirectly connected to other nodes in a network (i.e., the inverse of the sum of the distances). Given the presence of both positive and negative correlations within the network, we opted for the EI measure to identify the most influential symptoms with the highest EI (25). Symptoms were considered influential if their EI value lay above the 80th percentile.

#### Bridge network model

In bridge network analysis, we removed all within-cluster edges of the general network so that only the connections between the independent disorder clusters remained. We employed the “networktools” package to reveal key connectors that link the comorbid anxiety and IA network (Cramer et al., 2010). Within this bridge network, we computed bridge centrality indices—Bridge Expected Influence (BEI), Bridge Strength, Bridge Closeness, and Bridge Betweenness—similar to those in the general network. Because BEI incorporates both the magnitude and the sign of the associations, we used it as the primary indicator of a symptom’s bridging importance. These key connecting symptoms were identified by applying an 80th percentile cut-off to BEI values (42). The symptoms serve as potential targets for precision medicine to address the comorbidity of anxiety and IA.

#### Network stability analysis

For assessing conventional network model reliability and stability, the “bootnet” package was applied (43). We calculated a correlation stability coefficient (*CS coefficient*) for these centrality, bridge centrality, and EW indices in networks, showing how much data could be dropped while keeping a correlation of at least 0.70, which requires a *CS coefficient* of at least 0.25; results above 0.5 indicate strong network stability (43). Besides, with 1,000 case-dropping bootstrap simulations to estimate *95% confidence intervals (95% CIs)* for EI, BEI, and EW, non-overlapping *95% CIs* indicated significant differences between two symptoms or two edges (43).

#### Network comparison test

We explored differences in the combined anxiety-IA networks between rural and urban adolescents using the “Network ComparisonTest” package, based on 1,000 bootstrap samples, with Benjamini–Hochberg adjustment (44). The global strength differences test evaluated whether the overall network topology differs between rural and urban adolescents by comparing the sum of weights of all edges connected to that node between groups. The maximum EW differences test identified the single edge with the largest absolute weight difference between the two groups and used this difference as the test statistic for the overall structural invariance test.

We also subsequently fitted dedicated general and bridge networks for the rural and urban subsamples. For each residence-specific network, we estimated the structure with EBICglasso, quantified accuracy and stability via 1,000 non-parametric bootstraps, and identified the most influential symptoms and key connectors.

## Results

### Demographics and symptom distributions

The study included 60,268 adolescents aged 12 – 19 years (mean age: 15.90 ± 1.65 years; 54.44% females; 40.08% junior high school students; and 88.7% Han ethnicity), stratified by residence: rural (*n* = 49,819) and urban (*n* = 10,449). Demographic characteristics are presented in **Table 1**.

**Table 1:** Demographic characteristics and differences among groups by residence.

Variable	Total (*N* = 60,268)	Rural (*N* = 49,819)	Urban (*N* = 10,449)	*T/ X^2^/W*	*Degrees of freedom (df)*	*P value*
Age (years)	15.90 ± 1.65	15.92 ± 1.65	15.84 ± 1.65	4.443	15139	< .001 ***
Gender				0.002	1	.962
Female	32,809 (54.44%)	22,701 (45.57%)	4,758 (45.54%)			
Male	27,459 (45.56%)	27,118 (54.43%)	5,691 (54.46%)			
Grade				0.144	1	.705
Junior high school	24,157 (40.08%)	19,951 (40.05%)	4,206 (40.25%)			
Senior high school	36,111 (59.92%)	29,868 (59.95%)	6,243 (59.75%)			
Ethnicity				150.569	1	< .001 ***
Han	53,459 (88.70%)	43,829 (87.98%)	9,630 (92.16%)			
Others	6,809 (11.30%)	5,990 (12.02%)	819 (7.84%)			
Academic satisfaction				261204653	/	.524
Very satisfied	4,078 (6.77%)	3,375 (6.77%)	703 (6.73%)			
Satisfied	9,307 (15.44%)	7,502 (15.06%)	1,805 (17.27%)			
Neutral	34,422 (57.11%)	28,769 (57.75%)	5,653 (54.10%)			
Dissatisfied	10,085 (16.73%)	8,258 (16.58%)	1,827 (17.48%)			
Very dissatisfied	2,376 (3.94%)	1,915 (3.84%)	461 (4.41%)			
Single-child				1897.652	1	< .001 ***
No	45,252 (75.08%)	39,158 (78.60%)	4,355 (41.68%)			
Yes	15,016 (24.92%)	10,661 (21.40%)	6,094 (58.32%)			
Family income monthly (RMB ￥)				188153574	/	< .001 ***
<2,040	12,034 (19.97%)	10,868 (21.81%)	1,166 (11.16%)			
2,040-4,999	22,215 (36.86%)	19,383 (38.91%)	2,832 (27.10%)			
5,000-9,999	16,679 (27.67%)	13,280 (26.66%)	3,399 (32.53%)			
≥10,000	9,340 (15.50%)	6,288 (12.62%)	3,052 (29.21%)			
Marital status of parents				84.636	4	< .001 ***
Unmarried	1,033 (1.71%)	882 (1.77%)	151 (1.45%)			
Married	48,496 (80.47%)	40,324 (80.94%)	8,172 (78.21%)			
Divorced	5,615 (9.31%)	4,446 (8.92%)	1,169 (11.19%)			
Remarried	4,387 (7.28%)	3,528 (7.08%)	859 (8.22%)			
Other	737 (1.22%)	639 (1.28%)	98 (0.94%)			
Parenting style				113.754	3	< .001 ***
Authoritative	34,207 (56.76%)	27,793 (55.79%)	6,414 (61.38%)			
Autocratic	13,672 (22.69%)	11,580 (23.24%)	2,092 (20.02%)			
Ignorant	3,844 (6.38%)	3,208 (6.44%)	636 (6.09%)			
Submissive	8,545 (14.18%)	7,238 (14.53%)	1,307 (12.51%)			

***P value <.001.

In the GAD - 7, “Nervousness” (GAD - 1) emerged as the most severe reported (0.54 ± 0.78). In the IAT - 20, “Complained about being on the web for very long” (IAT5) emerged as the predominant symptom (2.80 ± 1.22). The total score for the GAD - 7 Scale was 3.52 ± 5.03, showcasing the severity of the anxiety symptoms cluster. The composite score for the IAT - 20 Scale stood at 44.75 ± 17.07, reflecting a substantial severity of the IA symptoms cluster. Both total scores of the scales differed significantly between urban and rural adolescents (in GAD - 7, *p* = .001; in IAT - 20, *p* <.001), underscoring the need for residence-stratified analyses. **Table 2** delineates the mean values and standard deviations (SDs) for the symptoms and overall scores of the GAD - 7 and IAT - 20 scales, with the details of the IAT - 17 scale in **Supplementary Table S1** in the **Supplementary Material**.

**Table 2:** Description and differences of the symptoms and overall scales of GAD-7 and IAT-20.

Symptom / Scale	Item content	Total	Rural	Urban	T	df	P value
**GAD1**	Nervousness	0.54 ± 0.78	0.54 ± 0.78	0.53 ± 0.79	1.010	14968	.313
**GAD2**	Uncontrollable worry	0.48 ± 0.78	0.48 ± 0.77	0.46 ± 0.79	2.105	14996	.035 *
**GAD3**	Excessive worry	0.54 ± 0.81	0.54 ± 0.80	0.53 ± 0.82	1.953	14992	.051
**GAD4**	Trouble relaxing	0.50 ± 0.79	0.50 ± 0.79	0.48 ± 0.79	3.253	15164	.001 **
**GAD5**	Restlessness	0.42 ± 0.74	0.43 ± 0.74	0.39 ± 0.72	5.345	15382	< .001 ***
**GAD6**	Irritability	0.51 ± 0.80	0.52 ± 0.80	0.50 ± 0.81	2.200	15025	.028 *
**GAD7**	Feeling afraid	0.52 ± 0.81	0.53 ± 0.81	0.48 ± 0.80	5.305	15315	< .001 ***
**IAT1**	Stay on the web beyond schedule	2.68 ± 1.13	2.68 ± 1.12	2.69 ± 1.16	-0.794	14848	.427
**IAT2**	Neglect household chores	2.48 ± 1.15	2.48 ± 1.14	2.48 ± 1.17	0.179	14960	.858
**IAT3**	Prefer Internet to intimacy	2.08 ± 1.14	2.09 ± 1.14	2.03 ± 1.14	5.197	15164	< .001 ***
**IAT4**	Form new online relationships	2.11 ± 1.11	2.11 ± 1.11	2.08 ± 1.15	2.922	14832	.003 **
**IAT5**	Complained about being on the web for very long	2.80 ± 1.22	2.80 ± 1.21	2.80 ± 1.24	0.410	14956	.682
**IAT6**	School work suffers	2.46 ± 1.16	2.47 ± 1.15	2.42 ± 1.17	3.583	14967	< .001 ***
**IAT7**	Check email first	2.06 ± 1.13	2.08 ± 1.13	1.98 ± 1.13	7.874	15188	< .001 ***
**IAT8**	Job performance suffers	2.05 ± 1.11	2.07 ± 1.11	1.99 ± 1.11	6.307	15141	< .001 ***
**IAT9**	Defensive or secretive about being on the web	2.70 ± 1.31	2.70 ± 1.31	2.68 ± 1.30	1.928	15175	.054
**IAT10**	Use the web to escape from emotion	2.49 ± 1.28	2.49 ± 1.28	2.46 ± 1.30	2.610	14958	.009 **
**IAT11**	Craving for next Internet use	2.22 ± 1.18	2.22 ± 1.18	2.22 ± 1.20	0.003	14951	0.997
**IAT12**	Fear about boredom if offline	2.19 ± 1.20	2.20 ± 1.20	2.14 ± 1.20	4.571	15185	< .001 ***
**IAT13**	Annoyed at being interrupted	1.96 ± 1.09	1.96 ± 1.09	1.95 ± 1.09	1.110	15109	0.267
**IAT14**	Lose sleep	2.08 ± 1.16	2.09 ± 1.16	2.03 ± 1.18	4.040	14993	< .001 ***
**IAT15**	Fantasize about being on the web	2.13 ± 1.12	2.13 ± 1.11	2.13 ± 1.13	0.132	14980	.895
**IAT16**	Reluctant to be offline	2.21 ± 1.17	2.21 ± 1.17	2.23 ± 1.18	-1.966	14992	.049 *
**IAT17**	Fail to stop being on the web	2.19 ± 1.17	2.20 ± 1.17	2.15 ± 1.17	4.328	15126	< .001 ***
**IAT18**	Hidden web time	1.92 ± 1.10	1.92 ± 1.10	1.88 ± 1.10	3.700	15186	< .001 ***
**IAT19**	Prefer Internet to going out	2.08 ± 1.14	2.09 ± 1.14	2.03 ± 1.14	5.197	15164	< .001 ***
**IAT20**	Web makes you feel better	1.85 ± 1.10	1.87 ± 1.11	1.77 ± 1.08	7.941	15454	< .001 ***
**GAD-7 scale**	/	3.52 ± 5.03	3.55 ± 5.03	3.37 ± 5.03	3.281	15140	.001 **
**IAT-20 scale**	/	44.75 ± 17.07	44.88 ± 17.07	44.15 ± 17.01	3.992	15188	< .001 ***

GAD means generalized anxiety disorder. IAT means Internet addiction test. The GAD-7 scale is the 7-item Generalized Anxiety Disorder Scale. The IAT-20 scale is the original 20-item Young Internet Addiction Test. *: P value < 0.05; **: P value < 0.01; ***: P value < 0.001.

### Network stability analysis

The 1,000-case-dropping bootstrap confirmed that the general and bridge networks for the overall population, as well as rural and urban subgroups, revealed acceptable stability, as evidenced by all *CS coefficients* of 0.750 for the main indices, including *EI*, *BEI*, and *EW*, indicating that the estimates did not fluctuate markedly when portions of the sample were removed. **Supplementary Figure S1** presents the *CS coefficients* of all centrality, bridge centrality, and EW metrics in the **Supplementary Materials**.

The case-dropping difference test showed that most of the comparisons of main metrics between pairs of nodes or edges were statistically significant. **Supplementary Figure S2** displays the resulting *95% CIs* of all *metrics* in the **Supplementary Materials**; non-overlapping intervals denote significant differences, whereas overlapping intervals reflect non-significant differences.

### General network model

**Figure 1:**
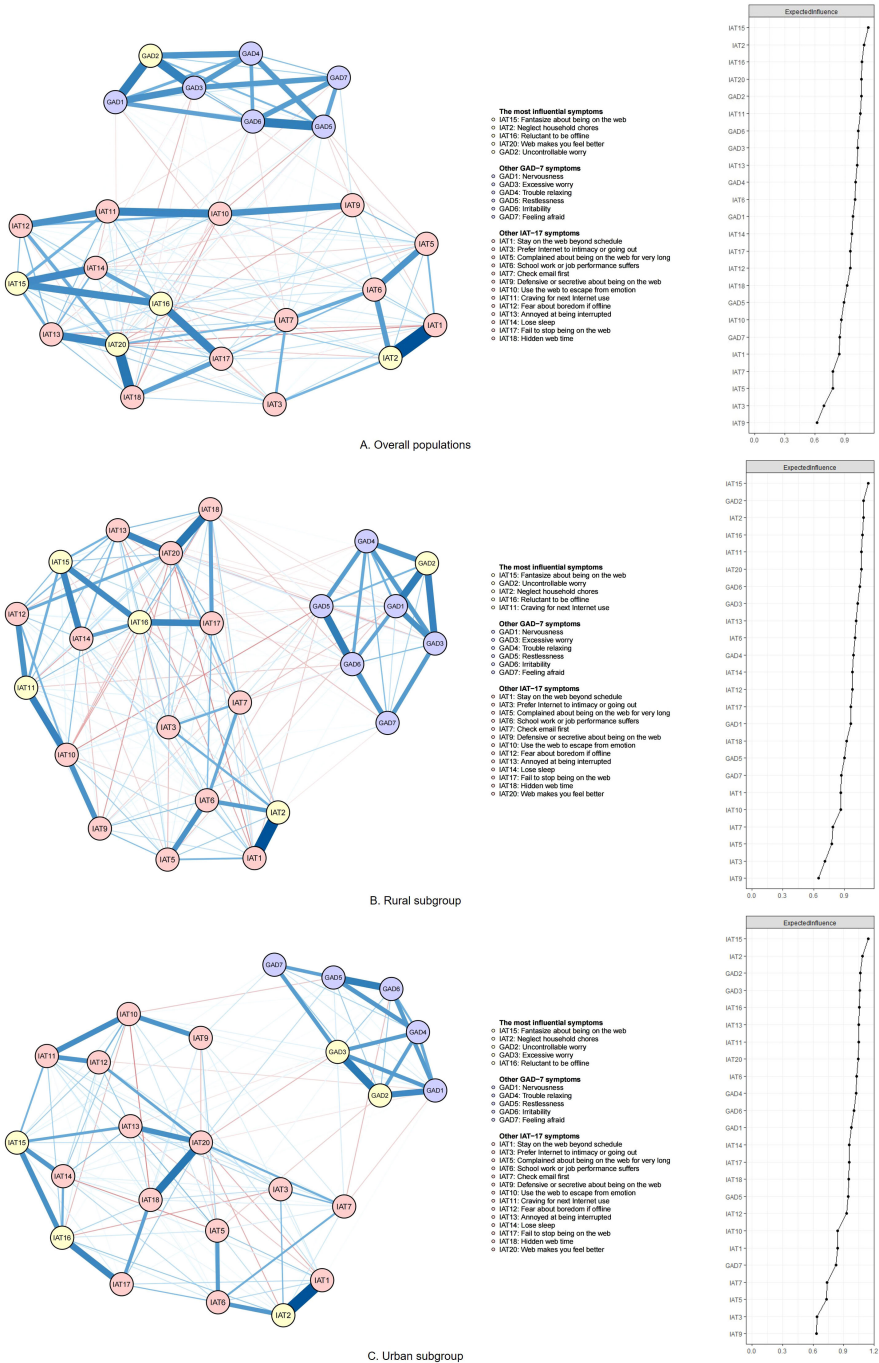
Network structure and expected influence (EI) value for each node of the combined anxiety-IA network. GAD - 7 means the 7-item Generalized Anxiety Disorder Scale. The IAT - 17 scale is the 17-item modified Internet Addiction Test. Nodes with EI values above the 80th percentile are identified as the most influential nodes. Blue lines represent positive correlations, while red lines indicate negative correlations. The edge’s thickness and saturation reflect the value of EI, with a higher absolute value of EI through greater thickness and saturation. **(A)** Overall populations; **(B)** Rural subgroup; **(C)** Urban subgroup.

**Figure 1** illustrates the general comorbid network structure with the EI values for each symptom. The most influential symptoms were “Fantasize about being on the web” (IAT15, *EI* = 1.136), “Neglect household chores” (IAT2, *EI* = 1.094), “Reluctant to be offline” (IAT16, *EI* = 1.071), “Web makes you feel better” (IAT20, EI = 1.067), and “Uncontrollable worry” (GAD2, *EI* = 1.066).

The strongest positive edges linked “Fantasize about being on the web” with “Neglect household chores” (IAT1-IAT2, *EW* = 0.406), followed by “Hidden web time” and “Web makes you feel better” (IAT18-IAT20, *EW* = 0.301) and “restlessness” and “irritability” (GAD5-GAD6, *EW* = 0.279).

The strongest negative connections were between “Fantasize about being on the web” and “Web makes you feel better” (IAT1-IAT20, *EW* = -0.048), “Use the web to escape from emotion” and “hidden web time” (IAT10-IAT18, *EW* = -0.038), and “Defensive or secretive about being on the web” and “Web makes you feel better” (IAT9-IAT20, *EW* = -0.035). The specific values of all EW and centrality metrics are listed in **Supplementary Tables S2** and **S3**, with the order in **Supplementary Figure S3** in the **Supplementary Materials**, respectively.

### Bridge network model

**Figure 2:**
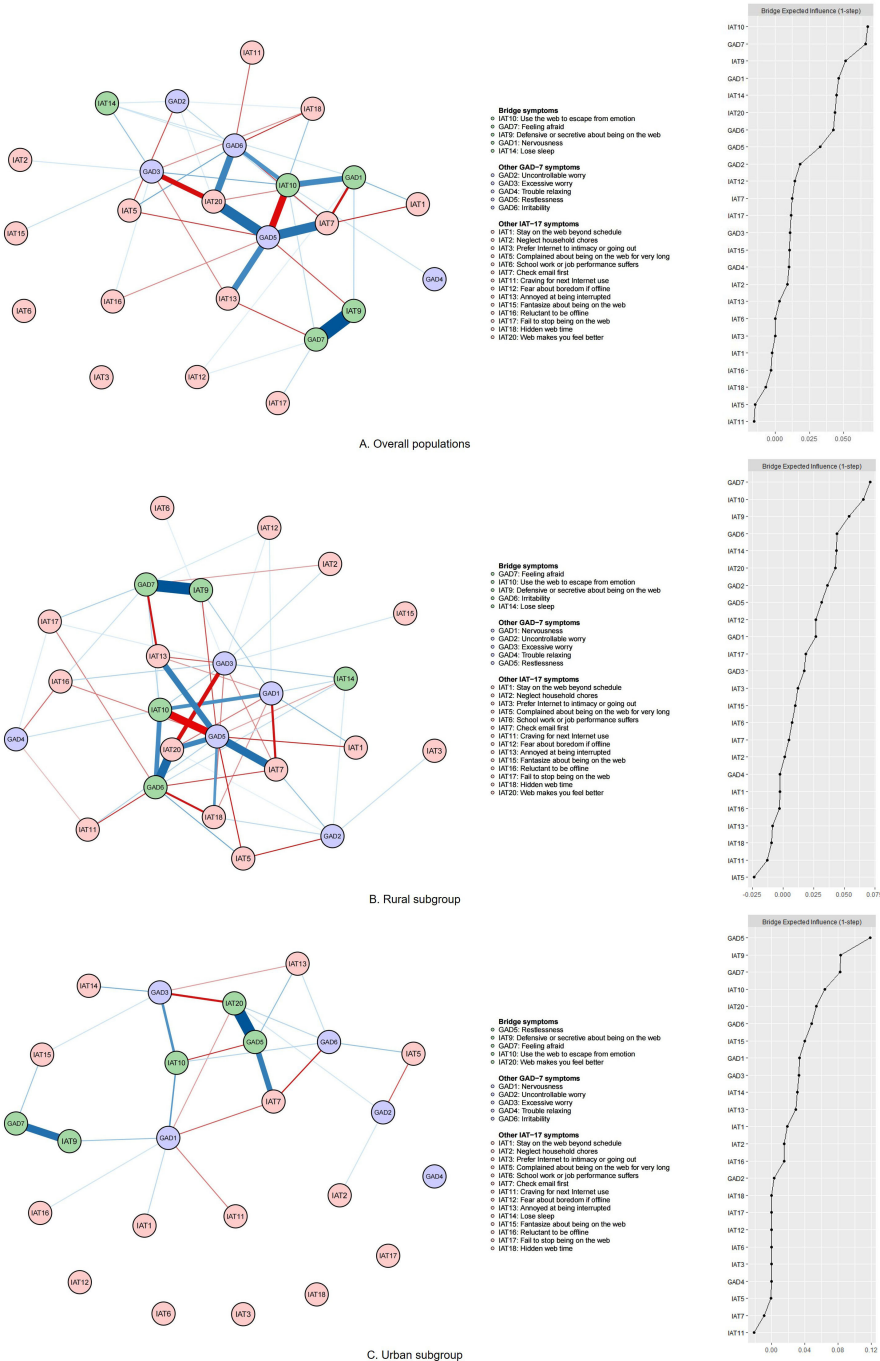
Bridge network structure and the bridge expected influence (BEI) value for each node of the combined anxiety and IA network. GAD-7 means the 7-item Generalized Anxiety Disorder Scale. The IAT-17 scale is the 17-item modified Internet Addiction Test. Nodes with BEI values above the 80th percentile are identified as bridge nodes. The item content, represented by node names, is displayed in the legend. Blue lines represent positive correlations, while red lines indicate negative correlations. The edge’s thickness and saturation reflect the value of EI, with a higher absolute value of EI through greater thickness and saturation. **(A)** Overall populations; **(B)** Rural subgroup; **(C)** Urban subgroup.

**Figure 2** highlights the key connectors that link anxiety and IA comorbidity, along with their BEI values. “Use the web to escape from emotion” (IAT10, *BEI* = 0.068), “Feeling afraid” (GAD7, *BEI* = 0.066), “Defensive or secretive about being on the web” (IAT9, *BEI* = 0.051), “Nervousness” (GAD1, *BEI* = 0.046), and “Lose sleep” (IAT14, *BEI* = 0.045) were identified as bridge nodes, all of which are important in connecting the comorbidity network.

The strongest positive bridge connected “Feeling afraid” with “Defensive or secretive about being on the web” (GAD7-IAT9, *EW* = 0.054), “Restlessness” with “Web makes you feel better” (GAD5-IAT20, *EW* = 0.044), and “Restlessness” with “Check email first” (GAD5-IAT7, *EW* = 0.042).

The strongest negative associations were “Restlessness” and “Use the web to escape from emotion” (GAD5-IAT10, *EW* = -0.035), “Excessive worry” and “Web makes you feel better” (GAD3-IAT20, *EW* = -0.031), and “Nervousness” and “Check email first” (GAD1-IAT7, *EW* = -0.024). The specific values of all bridge centrality metrics are seen in **Supplementary Table S3**, with the order in **Supplementary Figure S3** in the **Supplementary Materials**.

### Network comparison test

**Figure 3:**
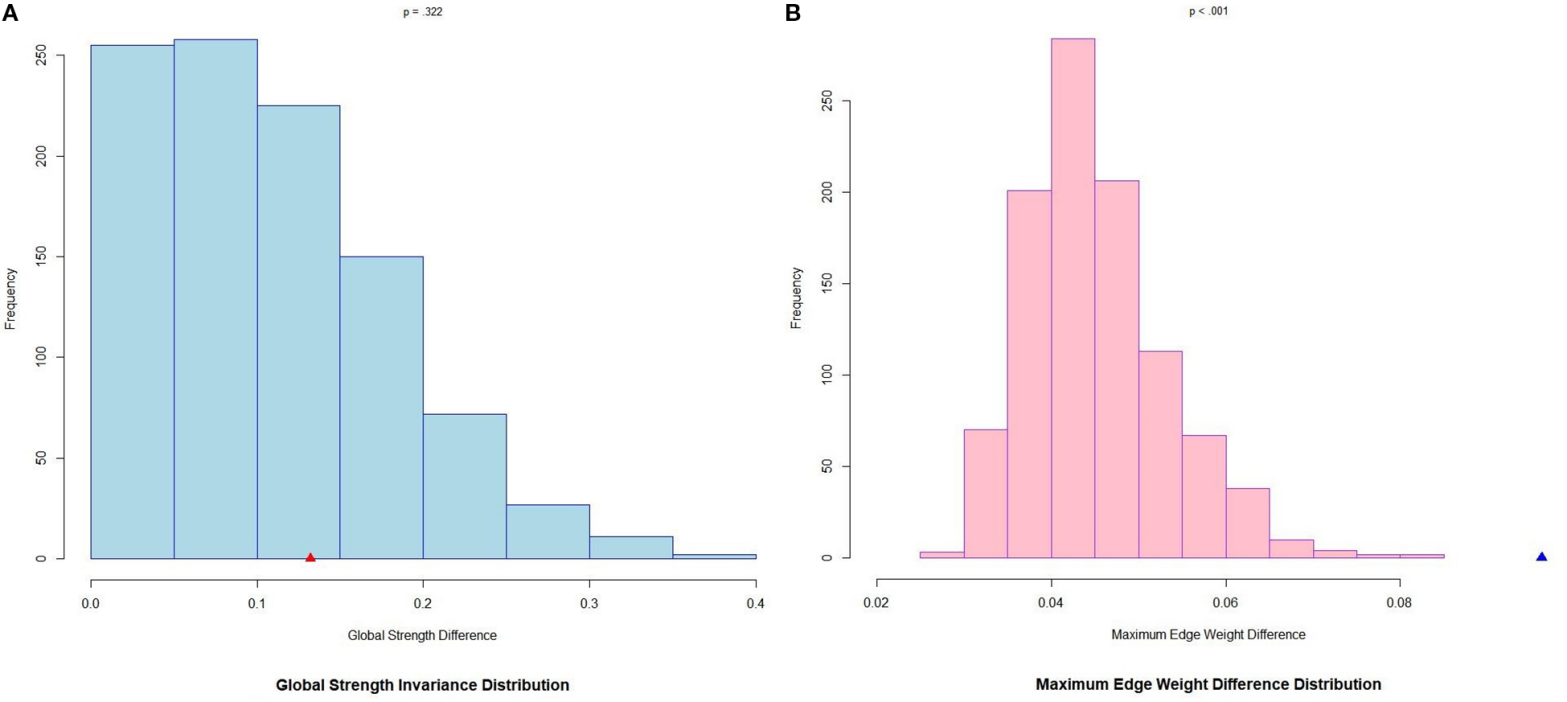
Global strength and edge weight comparisons between rural and urban networks of anxiety and IA symptoms. **(A)** Global strength (rural:11.844, urban:11.976) difference: point estimate of *Test Statistic S* = 0.132, p = .332. **(B)** Maximum edge weight difference: point estimate of *Test Statistic M* = 0.096, p <.001. Bars indicate the relative frequency of the test statistic under the null hypothesis of no group difference from 1,000 label-swapping permutations. The triangle denotes the observed point estimate from the original, unpermuted data. A triangle located far in the tail (outside the bars of the null distribution) signifies a significant group difference; a triangle falling within the bars of the distribution indicates that the observed difference is compatible with random fluctuation, and not statistically significant.

**Figure 3** presents the comparative analysis of the combined network models between rural and urban participants. The analysis of differences in global strength (rural: 11.844 vs. urban: 11.976) showed no significant differences between rural and urban participants (*Test Statistic S* = 4.834, *p* = .332), indicating the comparable overall symptom co-occurrence intensity between the two groups. However, the maximum EW difference test revealed resident differences in connectivity modal among adolescents (*Test Statistic M* = 0.096, *p* <.001). In other words, the entire network structure was not identical between rural and urban groups. However, there were no significant specific EW differences in the anxiety and IA combined network between rural and urban participants (all *p* >.05), as seen in **Supplementary Table S4** in the **Supplementary Materials**.

The most influential nodes and key bridge nodes differed between the two residence groups. In general networks, “Craving for next Internet use” (IAT11, *EI* = 1.062) only emerged as an influential symptom in rural adolescents, while “Excessive worry” (GAD3, *EI* = 1.061) only emerged as this symptom in urban adolescents. The residence-stratified groups shared the same strongest positive edges with the overall population (IAT1-IAT2, IAT18-IAT20, GAD5-GAD6). Notably, the most pronounced special negative edge in the rural network was linked to “Restlessness” with “Use the web to escape from emotion” (GAD5-IAT10, *EW* = -0.044). By contrast, the urban network exhibited two markedly strong negative connections: “Complained about being on the web for very long” and “Lose sleep” (IAT5-IAT14, *EW* = -0.056); and “Prefer Internet to intimacy or going out” and “Reluctant to be offline” (IAT3-IAT16, *EW* = -0.045).

In the bridge networks, there were key connectors only emerging in rural areas: “Irritability” (GAD6), with key connectors “Restlessness” (GAD5) and “Web makes you feel better” (IAT20) only emerging in urban areas. The different network models of the rural, urban, and overall populations shared the same strongest positive edge (GAD7-IAT9, GAD5-IAT20, GAD6-IAT20, and GAD5-IAT7). However, in urban adolescents, an additional strong negative edge emerged: “Irritability” and “Check email first” (GAD6-IAT7; *EW* = -0.035).

## Discussion

In the present study, we found “Fantasize about being on the web,” “Neglect house chores,” “Reluctant to be offline,” “Web makes you feel better,” and “Uncontrollable worry” emerged as the most influential symptoms in the combined anxiety-IA network, while “Use the web to escape from emotion,” “Feeling afraid,” “Defensive or secretive about being on the web,” “Nervousness,” and “Lose sleep” served as key bridge nodes linking the anxiety-IA network in adolescents. In addition, the most influential and bridging symptoms diverged by residence: “Craving for next Internet use” and “Irritability” characterized rural adolescents, whereas “Excessive worry,” “Restlessness,” and “Web makes you feel better” typified urban adolescents.

### General network model

“Fantasize about being on the web,” “Neglect household chores,” “Reluctant to be offline,” “Web makes you feel better,” and “Uncontrollable worry” emerged as the five most influential nodes, capturing intrusive cognition, functional impairment, withdrawal, emotional dependence, and the DSM - 5 core anxiety symptom, respectively. Specifically, fantasizing reflects intrusive daydreaming, which fuels IA and anxiety symptoms. It supports a previous study suggesting that day-to-day fluctuations in maladaptive daydreaming prospectively predicted simultaneous increases in negative emotion, whereas it also frequently co-occurs with anxiety and depression, indicating a positive-reinforcement loop akin to IA and anxiety (45). Neglecting chores is a highly visible marker of impairment of function for Chinese parents, who may routinely employ scolding and explicit expressions of disappointment—forms of psychological aggression and shaming discipline—to induce shame and guilt in their children when household duties or other filial expectations are neglected (46). In the context of emphasizing filial piety (47), these parental practices intensify children’s internalizing symptoms, creating a reciprocal loop in which anxiety and IA are both amplified. Reluctance to disconnect represents withdrawal and distress intolerance, reciprocally linked to physiological arousal (48) and fear-of-missing-out (49), thereby propagating symptoms rapidly across the anxiety-IA network. Feeling better embodies the reinforcement loop it creates—short-term mood repair by online immersion strengthens compulsive use, which then exacerbates offline stressors, such as schoolwork backlog and family tension, fueling new anxious conditions and motivation to IA (50). In addition to those, the result of uncontrollable worry aligns with previous adolescent comorbidity networks (51, 52) and confirms the criteria according to both *DSM-5* and *ICD-11* that “Uncontrollable worry” is one of the core symptoms of GAD (53, 54).

Except for these, fantasizing, reluctance, and feeling better might elucidate the fundamental neurobiological pathway implicated in IA. The “feels-better” pathway may activate the brain’s reward centers, such as the ventral striatum, illustrating how initial Internet use can offer pleasure or serve as an escape mechanism, potentially leading to addiction (55). Besides, the “must-do” pathway may become more prominent, influencing the dorsal striatum areas responsible for habit formation (55).

Furthermore, the results of this study showed that the strongest negative edge connected “Fantasize about being on the web” with “Web makes you feel better.” This inverse coupling suggests that the anticipatory craving and the consummatory reward do not co-activate; rather, they may represent distinct phases of the comorbidity cycle. These psychological dynamics highlight the probable transition from voluntary Internet use to a compulsive necessity, revealing the intricate relationship between reward, compulsion, and the development and persistence of IA and its comorbidity (50).

### Bridge network model

In the bridge network, the results of this study showed that “Use the web to escape from emotion,” “Feeling afraid,” “Defensive or secretive about being on the web,” “Nervousness,” and “Lose sleep” were identified as bridge nodes, important in connecting the comorbidity of anxiety and IA symptoms, capturing emotion regulation, transdiagnostic emotion, behavioral concealment, physiological arousal bridge, and biobehavior, respectively. Negative emotions may lead adolescents to adopt a protective stance towards their online activities, viewing the Internet as a refuge from external criticism or judgment, directly linking compensatory use of the Internet with anxiety symptoms (56). Besides, individuals prone to emotions such as feeling afraid and nervous exhibit sensitivity that may escalate into anxiety and IA comorbidity, supporting previous research findings that the generalized fear or nervousness intensifies the urge to seek online safety cues. Meanwhile, excessive Internet use feeds back into new offline fears—missed deadlines, parental scolding—which in turn intensify IA and anxiety symptoms, sustaining the comorbidity loop (12, 54). Hiding usage generates interpersonal tension and guilt (57), further binding the two disorders into a self-perpetuating network. Besides, this finding of losing sleep as a bridge node provided empirical support for the mechanisms by which sleep issues can affect both IA and anxiety symptoms (58, 59). Sleep deprivation can impair the brain’s capacity to regulate emotions effectively (59), adversely impact impulse control function (60), and negatively alter social interactions (61), which may ultimately reinforce patterns of IA and anxiety.

Interestingly, the most pronounced negative connection was between “Nervousness” and “Check email first.” This negative association may reflect a tendency among those who are generally nervous not to necessarily prioritize checking emails. This is also indicative of a larger trend among Chinese adolescents, who primarily use immediate, interactive digital platforms like WeChat rather than conventional email for communication (62).

### Network comparison test

The comparative analysis of the undirected combined networks between rural and urban adolescents revealed the insignificant influence of residence on these global network structures, which is consistent with previous research (27). However, conversely, the maximum EW difference test of the global network mode is significant.

In rural adolescents, the most influential symptoms, especially including “Craving for the next Internet use,” align with a network analysis of 1,009 Macua adolescents, in which the craving symptom was the central symptom linking IA and depression (63). Limited access to electronic devices in rural regions (64) is likely to amplify the salience of upcoming online rewards (65), because of which each opportunity to go online becomes highly valued. The heightened craving then triggers anxiety, reinforcing a feedback loop that makes craving the most efficient intervention target.

Notably, the most pronounced special negative edge in the rural bridge network was linked to “Restlessness” with “Use the web to escape from emotion,” indicating that when rural adolescents already feel physically agitated, they appear less inclined to turn to the Internet as an immediate emotional regulator. One plausible explanation is that the limited access to devices in rural settings makes online escape less available at the very moment restlessness peaks; instead, these adolescents may resort to non-digital coping (64, 65).

Excessive worry surfaced as a uniquely influential symptom of the general comorbid network only among urban adolescents. This may reflect the higher academic and social demands in cities (66), where unmet high-level needs become a central driver that simultaneously intensifies both anxiety and IA symptoms. The strongest negative edge linked complaints about prolonged Internet usage with losing sleep only in urban settings, where parental or school interventions intervene early and restrict late-night use of devices to protect sleep quality (67). On the other hand, the strongest negative edge connected a preference for online intimacy with reluctance to log off. For urban adolescents, digital closeness operates not as an irreplaceable emotional line but as an accessible form of entertainment (68), which can be turned off at will rather than feeling compelled to maintain.

The results of this study showed that restlessness and feeling better over the Internet served as bridge nodes in urban adolescents. Highly stressful city life constantly amplifies restlessness (66); when that restlessness is high, adolescents feel an immediate urge to go online, turning into a launching point for both anxiety and IA. The city-specific ecological and psychosocial factors—dense academic schedules (66) and high parental expectations (67)—make the “feels-better” pathway salient enough to function as a unique bridge between anxiety and IA symptoms, whereas rural adolescents—facing limited access and more stable routines (65)—do not experience this perception at comparable intensity. In addition to those, the strongest negative association includes a tendency among those who are generally irritable not to necessarily prioritize checking emails, indicating anger and the impulse to log on immediately do not rise together in urban adolescents.

### Clinical implications

Beyond these findings, our research suggests that when treating adolescent symptoms of anxiety and IA, it’s important to help them find alternative activities that provide comfort and pleasure, reducing the feelings of negative emotion they may experience when offline. Physicians should advise adolescents to engage in regular exercise and participate in social clubs, which can decrease their psychological reliance on the Internet. This approach not only offers a substitute for Internet surfing but can also aid in the recovery of their impaired brain and social functions (69). They should be trained in emotional skills as well, to prevent them from excessively seeking emotional solace or refuge online or from passively allowing their IA and anxiety symptoms to escalate (70).

### Limitations

However, the study had its limitations. The cross-sectional nature of the data collection limited our ability to draw rigorous causal links. Further longitudinal follow-up studies should be conducted to explore the causal relationship between anxiety and IA. Besides, the reliance on self-reported data and a sample limited to one province means that one should be cautious in generalizing these findings.

## Conclusions

Our study offers new insights into the symptom-level interactions between anxiety and IA in adolescents. The research points out how psychological dependence on the Internet can trigger comorbidity of anxiety and IA. It also highlights the need for good sleep practices to prevent comorbid IA and anxiety. In addition, for rural and urban adolescents, some differentiated specific interventions should be adopted. Larger-scale longitudinal studies are warranted to further explore these relationships and their treatment and prevention strategies.

## Data Availability

The raw data supporting the conclusions of this article will be made available by the authors, without undue reservation.
