# Supplementary material for: Symptom-level interactions between anxiety and internet addiction in Chinese adolescents: a large-scale network and residence difference analysis
Source: Front Psychiatry. 2025 Sep 16;16:1558276. doi: 10.3389/fpsyt.2025.1558276 (PMC12479496; doi:10.3389/fpsyt.2025.1558276)
Supplement: Supplementary file 1 [file DataSheet1.docx]

**Table S1**

Description and differences of the symptoms and overall scales of the modified IAT-17.

| **Symptom / Scale** | **Item content** | **Total** | **Rural** | **Urban** | **T** | **df** | **P value** |
| --- | --- | --- | --- | --- | --- | --- | --- |
| **IAT1** | Stay on the web beyond schedule | 2.68 ± 1.13 | 2.68 ± 1.12 | 2.69 ± 1.16 | -0.794 | 14848 | .427 |
| **IAT2** | Neglect household chores | 2.48 ± 1.15 | 2.48 ± 1.14 | 2.48 ± 1.17 | 0.179 | 14960 | .858 |
| **IAT3 (New)** | Prefer internet to intimacy or going out | 2.08 ± 1.14 | 2.09 ± 1.14 | 2.03 ± 1.14 | 5.197 | 15164 | ＜ .001 *** |
| **IAT4 (Deleted)** | / | / | / | / | / | / | / |
| **IAT5** | Complained about being on the web for very long | 2.80 ± 1.22 | 2.80 ± 1.21 | 2.80 ± 1.24 | 0.410 | 14956 | .682 |
| **IAT6 (New)** | School work or job performance suffers | 2.54 ± 1.16 | 2.55 ± 1.15 | 2.49 ± 1.18 | 4.481 | 14932 | ＜ .001 *** |
| **IAT7** | Check email first | 2.06 ± 1.13 | 2.08 ± 1.13 | 1.98 ± 1.13 | 7.874 | 15188 | ＜ .001 *** |
| **IAT8 (combined with IAT6)** | / | / | / | / | / | / | / |
| **IAT9** | Defensive or secretive about being on the web | 2.70 ± 1.31 | 2.70 ± 1.31 | 2.68 ± 1.30 | 1.928 | 15175 | .054 |
| **IAT10** | Use the web to escape from emotion | 2.49 ± 1.28 | 2.49 ± 1.28 | 2.46 ± 1.30 | 2.610 | 14958 | .009 ** |
| **IAT11** | Craving for next internet use | 2.22 ± 1.18 | 2.22 ± 1.18 | 2.22 ± 1.20 | 0.003 | 14951 | 0.997 |
| **IAT12** | Fear about boredom if offline | 2.19 ± 1.20 | 2.20 ± 1.20 | 2.14 ± 1.20 | 4.571 | 15185 | ＜ .001 *** |
| **IAT13** | Annoyed at being interrupted | 1.96 ± 1.09 | 1.96 ± 1.09 | 1.95 ± 1.09 | 1.110 | 15109 | 0.267 |
| **IAT14** | Lose sleep | 2.08 ± 1.16 | 2.09 ± 1.16 | 2.03 ± 1.18 | 4.040 | 14993 | ＜ .001 *** |
| **IAT15** | Fantasize about being on the web | 2.13 ± 1.12 | 2.13 ± 1.11 | 2.13 ± 1.13 | 0.132 | 14980 | .895 |
| **IAT16** | Reluctant to be offline | 2.21 ± 1.17 | 2.21 ± 1.17 | 2.23 ± 1.18 | -1.966 | 14992 | .049 * |
| **IAT17** | Fail to stop being on the web | 2.19 ± 1.17 | 2.20 ± 1.17 | 2.15 ± 1.17 | 4.328 | 15126 | ＜ .001 *** |
| **IAT18** | Hidden web time | 1.92 ± 1.10 | 1.92 ± 1.10 | 1.88 ± 1.10 | 3.700 | 15186 | ＜ .001 *** |
| **IAT19 (combined with IAT3)** | / | / | / | / | / | / | / |
| **IAT20** | Web makes you feel better | 1.85 ± 1.10 | 1.87 ± 1.11 | 1.77 ± 1.08 | 7.941 | 15454 | ＜ .001 *** |
| **IAT-17 scale** | / | 38.59 ± 14.83 | 38.68 ± 14.83 | 38.12± 14.82 | 3.560 | 15158 | ＜ .001 *** |

*Note:* The IAT-17 scale is the Khazaal 17-item modified Internet Addiction Test. This modification includes the removal of outmoded Item 4, the merging of repetitious Items 3 and 19, and the combination of repetitive Items 6 and 8 from the original 20-item Young Internet Addiction Test (IAT-20). For the combined items, the highest or only responses were used.

**Table S2A**

Edge weight of the anxiety and IA combined network of overall populations.

|  | **GAD1** | **GAD2** | **GAD3** | **GAD4** | **GAD5** | **GAD6** | **GAD7** | **IAT1** | **IAT2** | **IAT3** | **IAT5** | **IAT6** | **IAT7** | **IAT9** | **IAT10** | **IAT11** | **IAT12** | **IAT13** | **IAT14** | **IAT15** | **IAT16** | **IAT17** | **IAT18** | **IAT20** |
| --- | --- | --- | --- | --- | --- | --- | --- | --- | --- | --- | --- | --- | --- | --- | --- | --- | --- | --- | --- | --- | --- | --- | --- | --- |
| **GAD1** | 0.000 | 0.274 | 0.224 | 0.138 | 0.067 | 0.175 | 0.055 | 0.019 | 0.000 | 0.000 | 0.000 | 0.000 | -0.024 | 0.014 | 0.033 | 0.000 | 0.007 | 0.000 | 0.010 | 0.000 | 0.000 | 0.000 | 0.000 | -0.012 |
| **GAD2** | 0.274 | 0.000 | 0.270 | 0.215 | 0.116 | 0.093 | 0.079 | 0.000 | 0.000 | 0.000 | -0.017 | 0.000 | 0.012 | 0.000 | 0.000 | 0.000 | 0.000 | 0.000 | 0.009 | 0.000 | 0.000 | 0.000 | 0.007 | 0.008 |
| **GAD3** | 0.224 | 0.270 | 0.000 | 0.196 | 0.032 | 0.098 | 0.195 | 0.000 | 0.009 | 0.000 | 0.000 | 0.000 | 0.000 | 0.000 | 0.020 | 0.000 | 0.000 | -0.012 | 0.017 | 0.011 | 0.008 | 0.000 | -0.011 | -0.031 |
| **GAD4** | 0.138 | 0.215 | 0.196 | 0.000 | 0.195 | 0.155 | 0.098 | 0.000 | 0.000 | 0.000 | 0.000 | 0.000 | 0.000 | 0.000 | 0.010 | 0.000 | 0.000 | 0.000 | 0.000 | 0.000 | 0.000 | 0.000 | 0.000 | 0.000 |
| **GAD5** | 0.067 | 0.116 | 0.032 | 0.195 | 0.000 | 0.280 | 0.168 | -0.021 | 0.000 | 0.000 | -0.019 | 0.000 | 0.042 | -0.017 | -0.035 | 0.000 | 0.000 | 0.034 | 0.000 | 0.000 | -0.011 | 0.000 | 0.017 | 0.044 |
| **GAD6** | 0.175 | 0.093 | 0.098 | 0.155 | 0.280 | 0.000 | 0.192 | 0.000 | 0.000 | 0.000 | 0.021 | 0.000 | -0.017 | 0.000 | 0.029 | -0.015 | 0.000 | 0.000 | 0.009 | 0.000 | 0.000 | 0.000 | -0.019 | 0.035 |
| **GAD7** | 0.055 | 0.079 | 0.195 | 0.098 | 0.168 | 0.192 | 0.000 | 0.000 | 0.000 | 0.000 | 0.000 | 0.000 | 0.000 | 0.054 | 0.011 | 0.000 | 0.007 | -0.018 | 0.000 | 0.000 | 0.000 | 0.012 | 0.000 | 0.000 |
| **IAT1** | 0.019 | 0.000 | 0.000 | 0.000 | -0.021 | 0.000 | 0.000 | 0.000 | 0.406 | 0.065 | 0.114 | 0.080 | 0.030 | 0.053 | 0.064 | 0.000 | 0.000 | -0.030 | 0.050 | 0.000 | 0.012 | 0.061 | -0.011 | -0.048 |
| **IAT2** | 0.000 | 0.000 | 0.009 | 0.000 | 0.000 | 0.000 | 0.000 | 0.406 | 0.000 | 0.130 | 0.081 | 0.195 | 0.041 | 0.014 | 0.000 | 0.020 | 0.015 | 0.000 | 0.041 | 0.009 | 0.088 | 0.058 | 0.010 | -0.023 |
| **IAT3** | 0.000 | 0.000 | 0.000 | 0.000 | 0.000 | 0.000 | 0.000 | 0.065 | 0.130 | 0.000 | 0.019 | 0.013 | 0.134 | 0.000 | 0.000 | 0.050 | 0.053 | 0.081 | 0.028 | 0.022 | -0.021 | -0.022 | 0.064 | 0.072 |
| **IAT5** | 0.000 | -0.017 | 0.000 | 0.000 | -0.019 | 0.021 | 0.000 | 0.114 | 0.081 | 0.019 | 0.000 | 0.215 | 0.039 | 0.096 | 0.076 | 0.040 | 0.025 | 0.067 | -0.020 | 0.000 | 0.037 | 0.030 | 0.000 | -0.025 |
| **IAT6** | 0.000 | 0.000 | 0.000 | 0.000 | 0.000 | 0.000 | 0.000 | 0.080 | 0.195 | 0.013 | 0.215 | 0.000 | 0.154 | 0.078 | -0.013 | 0.014 | 0.024 | 0.000 | 0.055 | 0.000 | 0.050 | 0.106 | 0.031 | 0.000 |
| **IAT7** | -0.024 | 0.012 | 0.000 | 0.000 | 0.042 | -0.017 | 0.000 | 0.030 | 0.041 | 0.134 | 0.039 | 0.154 | 0.000 | 0.042 | 0.033 | 0.033 | 0.014 | 0.070 | 0.000 | 0.016 | -0.022 | 0.000 | 0.066 | 0.120 |
| **IAT9** | 0.014 | 0.000 | 0.000 | 0.000 | -0.017 | 0.000 | 0.054 | 0.053 | 0.014 | 0.000 | 0.096 | 0.078 | 0.042 | 0.000 | 0.221 | 0.033 | 0.000 | 0.000 | -0.019 | 0.021 | 0.000 | 0.053 | 0.015 | -0.035 |
| **IAT10** | 0.033 | 0.000 | 0.020 | 0.010 | -0.035 | 0.029 | 0.011 | 0.064 | 0.000 | 0.000 | 0.076 | -0.013 | 0.033 | 0.221 | 0.000 | 0.256 | 0.123 | 0.000 | 0.091 | 0.032 | -0.031 | 0.000 | -0.038 | -0.019 |
| **IAT11** | 0.000 | 0.000 | 0.000 | 0.000 | 0.000 | -0.015 | 0.000 | 0.000 | 0.020 | 0.050 | 0.040 | 0.014 | 0.033 | 0.033 | 0.256 | 0.000 | 0.225 | 0.092 | -0.024 | 0.138 | 0.089 | 0.034 | 0.048 | 0.022 |
| **IAT12** | 0.007 | 0.000 | 0.000 | 0.000 | 0.000 | 0.000 | 0.007 | 0.000 | 0.015 | 0.053 | 0.025 | 0.024 | 0.014 | 0.000 | 0.123 | 0.225 | 0.000 | 0.102 | 0.076 | 0.097 | 0.033 | 0.017 | -0.015 | 0.150 |
| **IAT13** | 0.000 | 0.000 | -0.012 | 0.000 | 0.034 | 0.000 | -0.018 | -0.030 | 0.000 | 0.081 | 0.067 | 0.000 | 0.070 | 0.000 | 0.000 | 0.092 | 0.102 | 0.000 | 0.110 | 0.136 | 0.050 | 0.000 | 0.093 | 0.252 |
| **IAT14** | 0.010 | 0.009 | 0.017 | 0.000 | 0.000 | 0.009 | 0.000 | 0.050 | 0.041 | 0.028 | -0.020 | 0.055 | 0.000 | -0.019 | 0.091 | -0.024 | 0.076 | 0.110 | 0.000 | 0.244 | 0.169 | 0.014 | 0.055 | 0.053 |
| **IAT15** | 0.000 | 0.000 | 0.011 | 0.000 | 0.000 | 0.000 | 0.000 | 0.000 | 0.009 | 0.022 | 0.000 | 0.000 | 0.016 | 0.021 | 0.032 | 0.138 | 0.097 | 0.136 | 0.244 | 0.000 | 0.238 | 0.061 | 0.031 | 0.080 |
| **IAT16** | 0.000 | 0.000 | 0.008 | 0.000 | -0.011 | 0.000 | 0.000 | 0.012 | 0.088 | -0.021 | 0.037 | 0.050 | -0.022 | 0.000 | -0.031 | 0.089 | 0.033 | 0.050 | 0.169 | 0.238 | 0.000 | 0.257 | 0.095 | 0.028 |
| **IAT17** | 0.000 | 0.000 | 0.000 | 0.000 | 0.000 | 0.000 | 0.012 | 0.061 | 0.058 | -0.022 | 0.030 | 0.106 | 0.000 | 0.053 | 0.000 | 0.034 | 0.017 | 0.000 | 0.014 | 0.061 | 0.257 | 0.000 | 0.181 | 0.094 |
| **IAT18** | 0.000 | 0.007 | -0.011 | 0.000 | 0.017 | -0.019 | 0.000 | -0.011 | 0.010 | 0.064 | 0.000 | 0.031 | 0.066 | 0.015 | -0.038 | 0.048 | -0.015 | 0.093 | 0.055 | 0.031 | 0.095 | 0.181 | 0.000 | 0.301 |
| **IAT20** | -0.012 | 0.008 | -0.031 | 0.000 | 0.044 | 0.035 | 0.000 | -0.048 | -0.023 | 0.072 | -0.025 | 0.000 | 0.120 | -0.035 | -0.019 | 0.022 | 0.150 | 0.252 | 0.053 | 0.080 | 0.028 | 0.094 | 0.301 | 0.000 |

**Table S2B**

Edge weight of the anxiety and IA combined network of rural participants.

|  | **GAD1** | **GAD2** | **GAD3** | **GAD4** | **GAD5** | **GAD6** | **GAD7** | **IAT1** | **IAT2** | **IAT3** | **IAT5** | **IAT6** | **IAT7** | **IAT9** | **IAT10** | **IAT11** | **IAT12** | **IAT13** | **IAT14** | **IAT15** | **IAT16** | **IAT17** | **IAT18** | **IAT20** |
| --- | --- | --- | --- | --- | --- | --- | --- | --- | --- | --- | --- | --- | --- | --- | --- | --- | --- | --- | --- | --- | --- | --- | --- | --- |
| **GAD1** | 0.000 | 0.278 | 0.225 | 0.130 | 0.077 | 0.167 | 0.055 | 0.020 | 0.000 | 0.000 | 0.000 | 0.000 | -0.027 | 0.016 | 0.032 | 0.000 | 0.009 | -0.011 | 0.014 | 0.000 | 0.000 | 0.000 | -0.011 | -0.015 |
| **GAD2** | 0.278 | 0.000 | 0.263 | 0.220 | 0.119 | 0.091 | 0.074 | 0.000 | 0.000 | 0.012 | -0.023 | 0.000 | 0.018 | 0.000 | 0.000 | 0.000 | 0.000 | 0.000 | 0.009 | 0.000 | 0.000 | 0.000 | 0.014 | 0.006 |
| **GAD3** | 0.225 | 0.263 | 0.000 | 0.195 | 0.040 | 0.098 | 0.192 | 0.000 | 0.012 | 0.000 | 0.000 | 0.007 | -0.012 | 0.000 | 0.016 | 0.000 | 0.008 | -0.017 | 0.017 | 0.010 | 0.015 | 0.008 | -0.014 | -0.033 |
| **GAD4** | 0.130 | 0.220 | 0.195 | 0.000 | 0.190 | 0.155 | 0.100 | 0.000 | 0.000 | 0.000 | 0.000 | 0.000 | 0.000 | 0.000 | 0.012 | -0.007 | 0.000 | 0.000 | 0.000 | 0.000 | -0.016 | 0.008 | 0.000 | 0.000 |
| **GAD5** | 0.077 | 0.119 | 0.040 | 0.190 | 0.000 | 0.279 | 0.163 | -0.022 | 0.000 | 0.000 | -0.023 | 0.000 | 0.045 | -0.019 | -0.044 | 0.015 | 0.000 | 0.039 | -0.010 | 0.000 | -0.014 | 0.000 | 0.029 | 0.036 |
| **GAD6** | 0.167 | 0.091 | 0.098 | 0.155 | 0.279 | 0.000 | 0.210 | 0.000 | 0.000 | 0.000 | 0.023 | 0.000 | -0.020 | 0.000 | 0.035 | -0.021 | 0.000 | 0.007 | 0.013 | 0.000 | 0.000 | -0.014 | -0.027 | 0.047 |
| **GAD7** | 0.055 | 0.074 | 0.192 | 0.100 | 0.163 | 0.210 | 0.000 | 0.000 | -0.010 | 0.000 | 0.000 | 0.000 | 0.000 | 0.057 | 0.015 | 0.000 | 0.009 | -0.027 | 0.000 | 0.000 | 0.011 | 0.016 | 0.000 | 0.000 |
| **IAT1** | 0.020 | 0.000 | 0.000 | 0.000 | -0.022 | 0.000 | 0.000 | 0.000 | 0.400 | 0.070 | 0.117 | 0.079 | 0.036 | 0.056 | 0.064 | 0.000 | 0.013 | -0.035 | 0.054 | 0.000 | 0.015 | 0.060 | -0.018 | -0.048 |
| **IAT2** | 0.000 | 0.000 | 0.012 | 0.000 | 0.000 | 0.000 | -0.010 | 0.400 | 0.000 | 0.132 | 0.082 | 0.190 | 0.039 | 0.013 | 0.000 | 0.026 | 0.014 | 0.000 | 0.042 | 0.012 | 0.084 | 0.059 | 0.011 | -0.026 |
| **IAT3** | 0.000 | 0.012 | 0.000 | 0.000 | 0.000 | 0.000 | 0.000 | 0.070 | 0.132 | 0.000 | 0.022 | 0.016 | 0.134 | 0.000 | 0.000 | 0.048 | 0.050 | 0.081 | 0.026 | 0.024 | -0.021 | -0.030 | 0.065 | 0.077 |
| **IAT5** | 0.000 | -0.023 | 0.000 | 0.000 | -0.023 | 0.023 | 0.000 | 0.117 | 0.082 | 0.022 | 0.000 | 0.217 | 0.039 | 0.097 | 0.081 | 0.036 | 0.027 | 0.060 | -0.020 | 0.000 | 0.035 | 0.031 | 0.000 | -0.028 |
| **IAT6** | 0.000 | 0.000 | 0.007 | 0.000 | 0.000 | 0.000 | 0.000 | 0.079 | 0.190 | 0.016 | 0.217 | 0.000 | 0.157 | 0.089 | -0.019 | 0.014 | 0.023 | 0.000 | 0.054 | -0.016 | 0.052 | 0.104 | 0.033 | 0.000 |
| **IAT7** | -0.027 | 0.018 | -0.012 | 0.000 | 0.045 | -0.020 | 0.000 | 0.036 | 0.039 | 0.134 | 0.039 | 0.157 | 0.000 | 0.041 | 0.039 | 0.031 | 0.011 | 0.073 | 0.000 | 0.023 | -0.028 | 0.000 | 0.071 | 0.114 |
| **IAT9** | 0.016 | 0.000 | 0.000 | 0.000 | -0.019 | 0.000 | 0.057 | 0.056 | 0.013 | 0.000 | 0.097 | 0.089 | 0.041 | 0.000 | 0.220 | 0.036 | 0.013 | 0.000 | -0.024 | 0.020 | 0.000 | 0.055 | 0.015 | -0.040 |
| **IAT10** | 0.032 | 0.000 | 0.016 | 0.012 | -0.044 | 0.035 | 0.015 | 0.064 | 0.000 | 0.000 | 0.081 | -0.019 | 0.039 | 0.220 | 0.000 | 0.262 | 0.120 | 0.000 | 0.095 | 0.030 | -0.036 | 0.000 | -0.036 | -0.026 |
| **IAT11** | 0.000 | 0.000 | 0.000 | -0.007 | 0.015 | -0.021 | 0.000 | 0.000 | 0.026 | 0.048 | 0.036 | 0.014 | 0.031 | 0.036 | 0.262 | 0.000 | 0.226 | 0.090 | -0.036 | 0.138 | 0.092 | 0.035 | 0.049 | 0.028 |
| **IAT12** | 0.009 | 0.000 | 0.008 | 0.000 | 0.000 | 0.000 | 0.009 | 0.013 | 0.014 | 0.050 | 0.027 | 0.023 | 0.011 | 0.013 | 0.120 | 0.226 | 0.000 | 0.105 | 0.077 | 0.097 | 0.032 | 0.016 | -0.023 | 0.149 |
| **IAT13** | -0.011 | 0.000 | -0.017 | 0.000 | 0.039 | 0.007 | -0.027 | -0.035 | 0.000 | 0.081 | 0.060 | 0.000 | 0.073 | 0.000 | 0.000 | 0.090 | 0.105 | 0.000 | 0.112 | 0.126 | 0.057 | 0.000 | 0.097 | 0.253 |
| **IAT14** | 0.014 | 0.009 | 0.017 | 0.000 | -0.010 | 0.013 | 0.000 | 0.054 | 0.042 | 0.026 | -0.020 | 0.054 | 0.000 | -0.024 | 0.095 | -0.036 | 0.077 | 0.112 | 0.000 | 0.251 | 0.174 | 0.018 | 0.058 | 0.054 |
| **IAT15** | 0.000 | 0.000 | 0.010 | 0.000 | 0.000 | 0.000 | 0.000 | 0.000 | 0.012 | 0.024 | 0.000 | -0.016 | 0.023 | 0.020 | 0.030 | 0.138 | 0.097 | 0.126 | 0.251 | 0.000 | 0.238 | 0.064 | 0.031 | 0.083 |
| **IAT16** | 0.000 | 0.000 | 0.015 | -0.016 | -0.014 | 0.000 | 0.011 | 0.015 | 0.084 | -0.021 | 0.035 | 0.052 | -0.028 | 0.000 | -0.036 | 0.092 | 0.032 | 0.057 | 0.174 | 0.238 | 0.000 | 0.253 | 0.098 | 0.034 |
| **IAT17** | 0.000 | 0.000 | 0.008 | 0.008 | 0.000 | -0.014 | 0.016 | 0.060 | 0.059 | -0.030 | 0.031 | 0.104 | 0.000 | 0.055 | 0.000 | 0.035 | 0.016 | 0.000 | 0.018 | 0.064 | 0.253 | 0.000 | 0.180 | 0.096 |
| **IAT18** | -0.011 | 0.014 | -0.014 | 0.000 | 0.029 | -0.027 | 0.000 | -0.018 | 0.011 | 0.065 | 0.000 | 0.033 | 0.071 | 0.015 | -0.036 | 0.049 | -0.023 | 0.097 | 0.058 | 0.031 | 0.098 | 0.180 | 0.000 | 0.299 |
| **IAT20** | -0.015 | 0.006 | -0.033 | 0.000 | 0.036 | 0.047 | 0.000 | -0.048 | -0.026 | 0.077 | -0.028 | 0.000 | 0.114 | -0.040 | -0.026 | 0.028 | 0.149 | 0.253 | 0.054 | 0.083 | 0.034 | 0.096 | 0.299 | 0.000 |

**Table S2C**

Edge weight of the anxiety and IA combined network of urban participants.

|  | **GAD1** | **GAD2** | **GAD3** | **GAD4** | **GAD5** | **GAD6** | **GAD7** | **IAT1** | **IAT2** | **IAT3** | **IAT5** | **IAT6** | **IAT7** | **IAT9** | **IAT10** | **IAT11** | **IAT12** | **IAT13** | **IAT14** | **IAT15** | **IAT16** | **IAT17** | **IAT18** | **IAT20** |
| --- | --- | --- | --- | --- | --- | --- | --- | --- | --- | --- | --- | --- | --- | --- | --- | --- | --- | --- | --- | --- | --- | --- | --- | --- |
| **GAD1** | 0.000 | 0.264 | 0.210 | 0.175 | 0.030 | 0.207 | 0.055 | 0.019 | 0.000 | 0.000 | 0.000 | 0.000 | -0.025 | 0.026 | 0.036 | -0.021 | 0.000 | 0.000 | 0.000 | 0.000 | 0.015 | 0.000 | 0.000 | -0.015 |
| **GAD2** | 0.264 | 0.000 | 0.312 | 0.192 | 0.095 | 0.098 | 0.100 | 0.000 | 0.015 | 0.000 | -0.026 | 0.000 | 0.000 | 0.000 | 0.000 | 0.000 | 0.000 | 0.000 | 0.000 | 0.000 | 0.000 | 0.000 | 0.000 | 0.014 |
| **GAD3** | 0.210 | 0.312 | 0.000 | 0.205 | 0.000 | 0.097 | 0.204 | 0.000 | 0.000 | 0.000 | 0.000 | 0.000 | 0.000 | 0.000 | 0.040 | 0.000 | 0.000 | -0.015 | 0.031 | 0.015 | 0.000 | 0.000 | 0.000 | -0.038 |
| **GAD4** | 0.175 | 0.192 | 0.205 | 0.000 | 0.219 | 0.150 | 0.084 | 0.000 | 0.000 | 0.000 | 0.000 | 0.000 | 0.000 | 0.000 | 0.000 | 0.000 | 0.000 | 0.000 | 0.000 | 0.000 | 0.000 | 0.000 | 0.000 | 0.000 |
| **GAD5** | 0.030 | 0.095 | 0.000 | 0.219 | 0.000 | 0.293 | 0.191 | 0.000 | 0.000 | 0.000 | 0.000 | 0.000 | 0.050 | 0.000 | -0.032 | 0.000 | 0.000 | 0.027 | 0.000 | 0.000 | 0.000 | 0.000 | 0.000 | 0.073 |
| **GAD6** | 0.207 | 0.098 | 0.097 | 0.150 | 0.293 | 0.000 | 0.111 | 0.000 | 0.000 | 0.000 | 0.026 | 0.000 | -0.035 | 0.000 | 0.020 | 0.000 | 0.000 | 0.017 | 0.000 | 0.000 | 0.000 | 0.000 | 0.000 | 0.020 |
| **GAD7** | 0.055 | 0.100 | 0.204 | 0.084 | 0.191 | 0.111 | 0.000 | 0.000 | 0.000 | 0.000 | 0.000 | 0.000 | 0.000 | 0.057 | 0.000 | 0.000 | 0.000 | 0.000 | 0.000 | 0.025 | 0.000 | 0.000 | 0.000 | 0.000 |
| **IAT1** | 0.019 | 0.000 | 0.000 | 0.000 | 0.000 | 0.000 | 0.000 | 0.000 | 0.428 | 0.053 | 0.099 | 0.080 | 0.000 | 0.043 | 0.067 | 0.000 | 0.000 | -0.031 | 0.045 | 0.000 | 0.000 | 0.077 | 0.000 | -0.042 |
| **IAT2** | 0.000 | 0.015 | 0.000 | 0.000 | 0.000 | 0.000 | 0.000 | 0.428 | 0.000 | 0.124 | 0.070 | 0.214 | 0.057 | 0.000 | 0.000 | 0.000 | 0.000 | 0.000 | 0.034 | 0.000 | 0.101 | 0.055 | 0.028 | -0.043 |
| **IAT3** | 0.000 | 0.000 | 0.000 | 0.000 | 0.000 | 0.000 | 0.000 | 0.053 | 0.124 | 0.000 | 0.000 | 0.000 | 0.129 | 0.000 | 0.000 | 0.067 | 0.065 | 0.081 | 0.041 | 0.000 | -0.045 | 0.000 | 0.071 | 0.054 |
| **IAT5** | 0.000 | -0.026 | 0.000 | 0.000 | 0.000 | 0.026 | 0.000 | 0.099 | 0.070 | 0.000 | 0.000 | 0.208 | 0.047 | 0.086 | 0.060 | 0.055 | 0.000 | 0.114 | -0.056 | 0.000 | 0.056 | 0.026 | 0.000 | -0.031 |
| **IAT6** | 0.000 | 0.000 | 0.000 | 0.000 | 0.000 | 0.000 | 0.000 | 0.080 | 0.214 | 0.000 | 0.208 | 0.000 | 0.146 | 0.039 | 0.000 | 0.032 | 0.029 | 0.000 | 0.080 | 0.000 | 0.058 | 0.118 | 0.023 | 0.000 |
| **IAT7** | -0.025 | 0.000 | 0.000 | 0.000 | 0.050 | -0.035 | 0.000 | 0.000 | 0.057 | 0.129 | 0.047 | 0.146 | 0.000 | 0.052 | 0.000 | 0.038 | 0.000 | 0.056 | 0.026 | 0.000 | 0.000 | 0.000 | 0.046 | 0.150 |
| **IAT9** | 0.026 | 0.000 | 0.000 | 0.000 | 0.000 | 0.000 | 0.057 | 0.043 | 0.000 | 0.000 | 0.086 | 0.039 | 0.052 | 0.000 | 0.231 | 0.000 | 0.000 | 0.000 | 0.000 | 0.043 | 0.000 | 0.052 | 0.040 | -0.035 |
| **IAT10** | 0.036 | 0.000 | 0.040 | 0.000 | -0.032 | 0.020 | 0.000 | 0.067 | 0.000 | 0.000 | 0.060 | 0.000 | 0.000 | 0.231 | 0.000 | 0.250 | 0.128 | 0.000 | 0.097 | 0.044 | -0.037 | 0.000 | -0.062 | 0.000 |
| **IAT11** | -0.021 | 0.000 | 0.000 | 0.000 | 0.000 | 0.000 | 0.000 | 0.000 | 0.000 | 0.067 | 0.055 | 0.032 | 0.038 | 0.000 | 0.250 | 0.000 | 0.225 | 0.090 | 0.000 | 0.140 | 0.091 | 0.024 | 0.057 | 0.000 |
| **IAT12** | 0.000 | 0.000 | 0.000 | 0.000 | 0.000 | 0.000 | 0.000 | 0.000 | 0.000 | 0.065 | 0.000 | 0.029 | 0.000 | 0.000 | 0.128 | 0.225 | 0.000 | 0.096 | 0.078 | 0.095 | 0.045 | 0.000 | 0.000 | 0.168 |
| **IAT13** | 0.000 | 0.000 | -0.015 | 0.000 | 0.027 | 0.017 | 0.000 | -0.031 | 0.000 | 0.081 | 0.114 | 0.000 | 0.056 | 0.000 | 0.000 | 0.090 | 0.096 | 0.000 | 0.110 | 0.169 | 0.021 | 0.000 | 0.072 | 0.243 |
| **IAT14** | 0.000 | 0.000 | 0.031 | 0.000 | 0.000 | 0.000 | 0.000 | 0.045 | 0.034 | 0.041 | -0.056 | 0.080 | 0.026 | 0.000 | 0.097 | 0.000 | 0.078 | 0.110 | 0.000 | 0.222 | 0.154 | 0.000 | 0.052 | 0.041 |
| **IAT15** | 0.000 | 0.000 | 0.015 | 0.000 | 0.000 | 0.000 | 0.025 | 0.000 | 0.000 | 0.000 | 0.000 | 0.000 | 0.000 | 0.043 | 0.044 | 0.140 | 0.095 | 0.169 | 0.222 | 0.000 | 0.241 | 0.044 | 0.035 | 0.071 |
| **IAT16** | 0.015 | 0.000 | 0.000 | 0.000 | 0.000 | 0.000 | 0.000 | 0.000 | 0.101 | -0.045 | 0.056 | 0.058 | 0.000 | 0.000 | -0.037 | 0.091 | 0.045 | 0.021 | 0.154 | 0.241 | 0.000 | 0.272 | 0.082 | 0.000 |
| **IAT17** | 0.000 | 0.000 | 0.000 | 0.000 | 0.000 | 0.000 | 0.000 | 0.077 | 0.055 | 0.000 | 0.026 | 0.118 | 0.000 | 0.052 | 0.000 | 0.024 | 0.000 | 0.000 | 0.000 | 0.044 | 0.272 | 0.000 | 0.190 | 0.096 |
| **IAT18** | 0.000 | 0.000 | 0.000 | 0.000 | 0.000 | 0.000 | 0.000 | 0.000 | 0.028 | 0.071 | 0.000 | 0.023 | 0.046 | 0.040 | -0.062 | 0.057 | 0.000 | 0.072 | 0.052 | 0.035 | 0.082 | 0.190 | 0.000 | 0.314 |
| **IAT20** | -0.015 | 0.014 | -0.038 | 0.000 | 0.073 | 0.020 | 0.000 | -0.042 | -0.043 | 0.054 | -0.031 | 0.000 | 0.150 | -0.035 | 0.000 | 0.000 | 0.168 | 0.243 | 0.041 | 0.071 | 0.000 | 0.096 | 0.314 | 0.000 |

**Table S3A**

The specific value of the influential indices of symptoms in the anxiety and IA combined network among overall populations.

|  | **Expected Influence** | **Strength** | **Closeness** | **Betweenness** | **Bridge Expected Influence (1-Step)** | **Bridge Expected Influence (2-Step)** | **Bridge Strength** | **Bridge Closeness** | **Bridge Betweenness** |
| --- | --- | --- | --- | --- | --- | --- | --- | --- | --- |
| **GAD1** | 0.980 | 1.053 | 0.001 | 2.000 | 0.046 | 0.046 | 0.119 | 0.008 | 17.000 |
| **GAD2** | 1.066 | 1.100 | 0.001 | 0.000 | 0.018 | 0.018 | 0.052 | 0.004 | 0.000 |
| **GAD3** | 1.027 | 1.135 | 0.002 | 10.000 | 0.011 | 0.011 | 0.119 | 0.007 | 19.000 |
| **GAD4** | 1.008 | 1.008 | 0.002 | 3.000 | 0.010 | 0.010 | 0.010 | 0.005 | 0.000 |
| **GAD5** | 0.891 | 1.097 | 0.002 | 52.000 | 0.033 | 0.033 | 0.239 | 0.007 | 20.000 |
| **GAD6** | 1.035 | 1.139 | 0.002 | 10.000 | 0.042 | 0.042 | 0.146 | 0.008 | 33.000 |
| **GAD7** | 0.852 | 0.889 | 0.002 | 30.000 | 0.066 | 0.066 | 0.103 | 0.006 | 6.000 |
| **IAT1** | 0.844 | 1.064 | 0.002 | 2.000 | -0.002 | -0.002 | 0.040 | 0.007 | 0.000 |
| **IAT2** | 1.094 | 1.139 | 0.002 | 14.000 | 0.009 | 0.009 | 0.009 | 0.005 | 0.000 |
| **IAT3** | 0.689 | 0.774 | 0.002 | 2.000 | 0.000 | 0.000 | 0.000 | NA | 0.000 |
| **IAT5** | 0.782 | 0.941 | 0.002 | 0.000 | -0.014 | -0.014 | 0.057 | 0.007 | 0.000 |
| **IAT6** | 1.003 | 1.028 | 0.002 | 28.000 | 0.000 | 0.000 | 0.000 | NA | 0.000 |
| **IAT7** | 0.782 | 0.908 | 0.002 | 24.000 | 0.013 | 0.013 | 0.095 | 0.008 | 5.000 |
| **IAT9** | 0.625 | 0.768 | 0.002 | 37.000 | 0.051 | 0.051 | 0.086 | 0.007 | 3.000 |
| **IAT10** | 0.864 | 1.134 | 0.002 | 35.000 | 0.068 | 0.068 | 0.138 | 0.012 | 53.000 |
| **IAT11** | 1.055 | 1.134 | 0.002 | 13.000 | -0.015 | -0.015 | 0.015 | NA | 0.000 |
| **IAT12** | 0.953 | 0.983 | 0.002 | 8.000 | 0.014 | 0.014 | 0.014 | 0.004 | 0.000 |
| **IAT13** | 1.025 | 1.146 | 0.002 | 13.000 | 0.003 | 0.003 | 0.064 | 0.007 | 0.000 |
| **IAT14** | 0.969 | 1.094 | 0.002 | 0.000 | 0.045 | 0.045 | 0.045 | 0.007 | 6.000 |
| **IAT15** | 1.136 | 1.136 | 0.002 | 4.000 | 0.011 | 0.011 | 0.011 | 0.005 | 0.000 |
| **IAT16** | 1.071 | 1.240 | 0.002 | 13.000 | -0.003 | -0.003 | 0.019 | 0.004 | 0.000 |
| **IAT17** | 0.957 | 1.000 | 0.002 | 18.000 | 0.012 | 0.012 | 0.012 | 0.004 | 0.000 |
| **IAT18** | 0.922 | 1.111 | 0.002 | 16.000 | -0.007 | -0.007 | 0.054 | 0.006 | 0.000 |
| **IAT20** | 1.067 | 1.452 | 0.002 | 51.000 | 0.044 | 0.044 | 0.130 | 0.010 | 28.000 |

**Table S3B**

The specific value of the influential indices of symptoms in the anxiety and IA combined network among rural participants.

|  | **Expected Influence** | **Strength** | **Closeness** | **Betweenness** | **Bridge Expected Influence (1-Step)** | **Bridge Expected Influence (2-Step)** | **Bridge Strength** | **Bridge Closeness** | **Bridge Betweenness** |
| --- | --- | --- | --- | --- | --- | --- | --- | --- | --- |
| **GAD1** | 0.959 | 1.087 | 0.002 | 6.000 | 0.027 | 0.027 | 0.154 | 0.007 | 12.000 |
| **GAD2** | 1.081 | 1.128 | 0.002 | 0.000 | 0.036 | 0.036 | 0.083 | 0.006 | 6.000 |
| **GAD3** | 1.029 | 1.182 | 0.002 | 8.000 | 0.017 | 0.017 | 0.170 | 0.008 | 23.000 |
| **GAD4** | 0.987 | 1.033 | 0.002 | 3.000 | -0.003 | -0.003 | 0.043 | 0.005 | 0.000 |
| **GAD5** | 0.899 | 1.165 | 0.002 | 34.000 | 0.032 | 0.032 | 0.297 | 0.007 | 30.000 |
| **GAD6** | 1.045 | 1.208 | 0.002 | 30.000 | 0.044 | 0.044 | 0.207 | 0.008 | 41.000 |
| **GAD7** | 0.866 | 0.940 | 0.002 | 23.000 | 0.071 | 0.071 | 0.146 | 0.007 | 9.000 |
| **IAT1** | 0.861 | 1.108 | 0.002 | 3.000 | -0.003 | -0.003 | 0.042 | 0.007 | 0.000 |
| **IAT2** | 1.081 | 1.153 | 0.002 | 13.000 | 0.001 | 0.001 | 0.022 | 0.005 | 0.000 |
| **IAT3** | 0.707 | 0.808 | 0.002 | 2.000 | 0.012 | 0.012 | 0.012 | 0.004 | 0.000 |
| **IAT5** | 0.773 | 0.963 | 0.002 | 0.000 | -0.024 | -0.024 | 0.070 | 0.008 | 0.000 |
| **IAT6** | 1.001 | 1.071 | 0.002 | 30.000 | 0.007 | 0.007 | 0.007 | 0.004 | 0.000 |
| **IAT7** | 0.786 | 0.961 | 0.002 | 26.000 | 0.005 | 0.005 | 0.123 | 0.009 | 11.000 |
| **IAT9** | 0.645 | 0.812 | 0.002 | 29.000 | 0.054 | 0.054 | 0.092 | 0.008 | 3.000 |
| **IAT10** | 0.861 | 1.183 | 0.002 | 36.000 | 0.066 | 0.066 | 0.154 | 0.014 | 61.000 |
| **IAT11** | 1.062 | 1.192 | 0.002 | 25.000 | -0.013 | -0.013 | 0.044 | 0.006 | 0.000 |
| **IAT12** | 0.977 | 1.024 | 0.002 | 7.000 | 0.027 | 0.027 | 0.027 | 0.006 | 0.000 |
| **IAT13** | 1.010 | 1.190 | 0.002 | 13.000 | -0.009 | -0.009 | 0.102 | 0.008 | 0.000 |
| **IAT14** | 0.978 | 1.157 | 0.002 | 0.000 | 0.043 | 0.043 | 0.063 | 0.009 | 8.000 |
| **IAT15** | 1.131 | 1.162 | 0.002 | 3.000 | 0.010 | 0.010 | 0.010 | 0.005 | 0.000 |
| **IAT16** | 1.075 | 1.305 | 0.002 | 11.000 | -0.003 | -0.003 | 0.056 | 0.006 | 0.000 |
| **IAT17** | 0.961 | 1.047 | 0.002 | 17.000 | 0.018 | 0.018 | 0.046 | 0.006 | 0.000 |
| **IAT18** | 0.920 | 1.176 | 0.002 | 16.000 | -0.010 | -0.010 | 0.094 | 0.008 | 0.000 |
| **IAT20** | 1.062 | 1.494 | 0.003 | 46.000 | 0.043 | 0.043 | 0.138 | 0.012 | 38.000 |

**Table S3C**

The specific value of the influential indices of symptoms in the anxiety and IA combined network among urban participants.

|  | **Expected Influence** | **Strength** | **Closeness** | **Betweenness** | **Bridge Expected Influence (1-Step)** | **Bridge Expected Influence (2-Step)** | **Bridge Strength** | **Bridge Closeness** | **Bridge Betweenness** |
| --- | --- | --- | --- | --- | --- | --- | --- | --- | --- |
| **GAD1** | 0.974 | 1.096 | 0.002 | 2.000 | 0.034 | 0.034 | 0.156 | 0.009 | 20.000 |
| **GAD2** | 1.063 | 1.116 | 0.002 | 0.000 | 0.003 | 0.003 | 0.056 | 0.006 | 5.000 |
| **GAD3** | 1.061 | 1.167 | 0.002 | 11.000 | 0.033 | 0.033 | 0.139 | 0.009 | 8.000 |
| **GAD4** | 1.025 | 1.025 | 0.002 | 25.000 | 0.000 | 0.000 | 0.000 | NA | 0.000 |
| **GAD5** | 0.945 | 1.009 | 0.002 | 81.000 | 0.119 | 0.119 | 0.182 | 0.008 | 6.000 |
| **GAD6** | 1.004 | 1.073 | 0.002 | 15.000 | 0.048 | 0.048 | 0.118 | 0.010 | 29.000 |
| **GAD7** | 0.827 | 0.827 | 0.002 | 9.000 | 0.082 | 0.082 | 0.082 | 0.007 | 1.000 |
| **IAT1** | 0.839 | 0.983 | 0.002 | 1.000 | 0.019 | 0.019 | 0.019 | 0.007 | 0.000 |
| **IAT2** | 1.083 | 1.169 | 0.002 | 14.000 | 0.015 | 0.015 | 0.015 | 0.005 | 0.000 |
| **IAT3** | 0.639 | 0.730 | 0.002 | 1.000 | 0.000 | 0.000 | 0.000 | NA | 0.000 |
| **IAT5** | 0.733 | 0.960 | 0.002 | 6.000 | 0.000 | 0.000 | 0.052 | 0.009 | 0.000 |
| **IAT6** | 1.027 | 1.027 | 0.002 | 26.000 | 0.000 | 0.000 | 0.000 | NA | 0.000 |
| **IAT7** | 0.736 | 0.856 | 0.003 | 30.000 | -0.009 | -0.009 | 0.110 | 0.008 | 0.000 |
| **IAT9** | 0.634 | 0.704 | 0.002 | 10.000 | 0.083 | 0.083 | 0.083 | 0.009 | 9.000 |
| **IAT10** | 0.841 | 1.103 | 0.002 | 20.000 | 0.064 | 0.064 | 0.127 | 0.013 | 34.000 |
| **IAT11** | 1.048 | 1.090 | 0.002 | 9.000 | -0.021 | -0.021 | 0.021 | NA | 0.000 |
| **IAT12** | 0.928 | 0.928 | 0.003 | 9.000 | 0.000 | 0.000 | 0.000 | NA | 0.000 |
| **IAT13** | 1.050 | 1.142 | 0.003 | 25.000 | 0.029 | 0.029 | 0.059 | 0.009 | 0.000 |
| **IAT14** | 0.957 | 1.069 | 0.002 | 0.000 | 0.031 | 0.031 | 0.031 | 0.008 | 0.000 |
| **IAT15** | 1.143 | 1.143 | 0.003 | 3.000 | 0.040 | 0.040 | 0.040 | 0.007 | 1.000 |
| **IAT16** | 1.055 | 1.219 | 0.002 | 13.000 | 0.015 | 0.015 | 0.015 | 0.006 | 0.000 |
| **IAT17** | 0.953 | 0.953 | 0.003 | 19.000 | 0.000 | 0.000 | 0.000 | NA | 0.000 |
| **IAT18** | 0.949 | 1.074 | 0.003 | 21.000 | 0.000 | 0.000 | 0.000 | NA | 0.000 |
| **IAT20** | 1.041 | 1.448 | 0.003 | 71.000 | 0.054 | 0.054 | 0.160 | 0.011 | 25.000 |

**Table S4**

No significant edge weight differences of the anxiety and IA combined network between rural and urban participants [showing p-value (Test Statistic E)].

|  | **GAD1** | **GAD2** | **GAD3** | **GAD4** | **GAD5** | **GAD6** | **GAD7** | **IAT1** | **IAT2** | **IAT3** | **IAT5** | **IAT6** | **IAT7** | **IAT9** | **IAT10** | **IAT11** | **IAT12** | **IAT13** | **IAT14** | **IAT15** | **IAT16** | **IAT17** | **IAT18** | **IAT20** |
| --- | --- | --- | --- | --- | --- | --- | --- | --- | --- | --- | --- | --- | --- | --- | --- | --- | --- | --- | --- | --- | --- | --- | --- | --- |
| **GAD1** | / | 1.000 (0.013) | 1.000 (0.015) | 0.454 (0.043) | 0.386 (0.046) | 0.518 (0.039) | 1.000 (0.000) | 1.000 (0.002) | 1.000 (0.002) | 1.000 (0.000) | 1.000 (0.000) | 0.577 (0.003) | 1.000 (0.002) | 1.000 (0.004) | 1.000 (0.006) | 0.386 (0.015) | 1.000 (0.001) | 1.000 (0.000) | 1.000 (0.002) | 1.000 (0.000) | 1.000 (0.000) | 0.914 (0.001) | 1.000 (0.006) | 1.000 (0.000) |
| **GAD2** | 1.000 (0.013) | / | 0.386 (0.049) | 0.814 (0.026) | 0.863 (0.025) | 1.000 (0.006) | 0.860 (0.025) | 1.000 (0.000) | 0.184 (0.012) | 0.769 (0.008) | 0.865 (0.003) | 0.165 (0.008) | 1.000 (0.000) | 1.000 (0.000) | 1.000 (0.000) | 1.000 (0.000) | 1.000 (0.005) | 1.000 (0.000) | 1.000 (0.000) | 1.000 (0.006) | 1.000 (0.000) | 1.000 (0.000) | 1.000 (0.000) | 1.000 (0.000) |
| **GAD3** | 1.000 (0.015) | 0.386 (0.049) | / | 1.000 (0.010) | 0.577 (0.035) | 1.000 (0.001) | 1.000 (0.011) | 1.000 (0.000) | 0.963 (0.008) | 1.000 (0.000) | 1.000 (0.000) | 1.000 (0.003) | 1.000 (0.000) | 1.000 (0.000) | 0.386 (0.024) | 1.000 (0.000) | 1.000 (0.002) | 1.000 (0.005) | 0.963 (0.012) | 1.000 (0.001) | 1.000 (0.000) | 0.982 (0.005) | 1.000 (0.005) | 1.000 (0.006) |
| **GAD4** | 0.454 (0.043) | 0.814 (0.026) | 1.000 (0.010) | / | 0.789 (0.028) | 1.000 (0.006) | 1.000 (0.016) | 1.000 (0.000) | 1.000 (0.000) | 1.000 (0.001) | 1.000 (0.000) | 0.769 (0.001) | 1.000 (0.000) | 1.000 (0.000) | 1.000 (0.002) | 1.000 (0.002) | 1.000 (0.000) | 1.000 (0.000) | 1.000 (0.000) | 0.814 (0.001) | 1.000 (0.000) | 1.000 (0.003) | 1.000 (0.000) | 1.000 (0.000) |
| **GAD5** | 0.386 (0.046) | 0.863 (0.025) | 0.577 (0.035) | 0.789 (0.028) | / | 1.000 (0.014) | 0.809 (0.027) | 1.000 (0.000) | 1.000 (0.000) | 1.000 (0.000) | 1.000 (0.001) | 1.000 (0.000) | 1.000 (0.001) | 1.000 (0.002) | 1.000 (0.009) | 1.000 (0.000) | 1.000 (0.000) | 1.000 (0.010) | 1.000 (0.000) | 1.000 (0.000) | 0.769 (0.005) | 1.000 (0.000) | 1.000 (0.001) | 0.514 (0.023) |
| **GAD6** | 0.518 (0.039) | 1.000 (0.006) | 1.000 (0.001) | 1.000 (0.006) | 1.000 (0.014) | / | 0.092 (0.096) | 1.000 (0.000) | 1.000 (0.000) | 1.000 (0.000) | 1.000 (0.000) | 1.000 (0.000) | 0.532 (0.008) | 1.000 (0.000) | 1.000 (0.012) | 1.000 (0.006) | 1.000 (0.006) | 1.000 (0.000) | 1.000 (0.002) | 0.368 (0.013) | 1.000 (0.000) | 1.000 (0.000) | 1.000 (0.000) | 0.733 (0.014) |
| **GAD7** | 1.000 (0.000) | 0.860 (0.025) | 1.000 (0.011) | 1.000 (0.016) | 0.809 (0.027) | 0.092 (0.096) | / | 1.000 (0.000) | 1.000 (0.000) | 1.000 (0.000) | 1.000 (0.000) | 1.000 (0.000) | 1.000 (0.000) | 1.000 (0.004) | 1.000 (0.009) | 1.000 (0.000) | 1.000 (0.004) | 1.000 (0.004) | 1.000 (0.000) | 0.514 (0.014) | 1.000 (0.000) | 1.000 (0.003) | 1.000 (0.000) | 1.000 (0.000) |
| **IAT1** | 1.000 (0.002) | 1.000 (0.000) | 1.000 (0.000) | 1.000 (0.000) | 1.000 (0.000) | 1.000 (0.000) | 1.000 (0.000) | / | 0.493 (0.027) | 0.865 (0.016) | 0.729 (0.020) | 1.000 (0.000) | 0.769 (0.019) | 0.901 (0.014) | 1.000 (0.000) | 1.000 (0.000) | 0.865 (0.008) | 1.000 (0.001) | 1.000 (0.012) | 1.000 (0.000) | 1.000 (0.001) | 0.758 (0.020) | 1.000 (0.000) | 1.000 (0.004) |
| **IAT2** | 1.000 (0.002) | 0.184 (0.012) | 0.963 (0.008) | 1.000 (0.000) | 1.000 (0.000) | 1.000 (0.000) | 1.000 (0.000) | 0.493 (0.027) | / | 1.000 (0.007) | 1.000 (0.012) | 0.631 (0.024) | 0.769 (0.019) | 1.000 (0.010) | 1.000 (0.000) | 0.631 (0.020) | 1.000 (0.008) | 1.000 (0.000) | 1.000 (0.004) | 1.000 (0.008) | 0.814 (0.018) | 1.000 (0.000) | 0.518 (0.015) | 0.532 (0.017) |
| **IAT3** | 1.000 (0.000) | 0.769 (0.008) | 1.000 (0.000) | 1.000 (0.001) | 1.000 (0.000) | 1.000 (0.000) | 1.000 (0.000) | 0.865 (0.016) | 1.000 (0.007) | / | 1.000 (0.007) | 1.000 (0.007) | 1.000 (0.005) | 1.000 (0.000) | 0.659 (0.014) | 0.758 (0.020) | 1.000 (0.012) | 1.000 (0.001) | 0.960 (0.015) | 1.000 (0.006) | 0.454 (0.018) | 1.000 (0.003) | 1.000 (0.009) | 0.769 (0.020) |
| **IAT5** | 1.000 (0.000) | 0.865 (0.003) | 1.000 (0.000) | 1.000 (0.000) | 1.000 (0.001) | 1.000 (0.000) | 1.000 (0.000) | 0.729 (0.020) | 1.000 (0.012) | 1.000 (0.007) | / | 1.000 (0.010) | 1.000 (0.006) | 1.000 (0.013) | 0.561 (0.023) | 0.643 (0.021) | 1.000 (0.012) | 0.092 (0.057) | 0.165 (0.026) | 0.532 (0.010) | 0.577 (0.022) | 1.000 (0.004) | 1.000 (0.000) | 1.000 (0.004) |
| **IAT6** | 0.577 (0.003) | 0.165 (0.008) | 1.000 (0.003) | 0.769 (0.001) | 1.000 (0.000) | 1.000 (0.000) | 1.000 (0.000) | 1.000 (0.000) | 0.631 (0.024) | 1.000 (0.007) | 1.000 (0.010) | / | 1.000 (0.008) | 0.092 (0.051) | 1.000 (0.000) | 0.631 (0.018) | 1.000 (0.003) | 1.000 (0.000) | 0.518 (0.025) | 1.000 (0.000) | 1.000 (0.008) | 0.901 (0.016) | 1.000 (0.008) | 1.000 (0.000) |
| **IAT7** | 1.000 (0.002) | 1.000 (0.000) | 1.000 (0.000) | 1.000 (0.000) | 1.000 (0.001) | 0.532 (0.008) | 1.000 (0.000) | 0.769 (0.019) | 0.769 (0.019) | 1.000 (0.005) | 1.000 (0.006) | 1.000 (0.008) | / | 1.000 (0.010) | 0.542 (0.024) | 1.000 (0.008) | 1.000 (0.011) | 0.963 (0.015) | 0.514 (0.023) | 0.814 (0.015) | 1.000 (0.000) | 1.000 (0.000) | 0.769 (0.020) | 0.276 (0.039) |
| **IAT9** | 1.000 (0.004) | 1.000 (0.000) | 1.000 (0.000) | 1.000 (0.000) | 1.000 (0.002) | 1.000 (0.000) | 1.000 (0.004) | 0.901 (0.014) | 1.000 (0.010) | 1.000 (0.000) | 1.000 (0.013) | 0.092 (0.051) | 1.000 (0.010) | / | 1.000 (0.009) | 0.809 (0.017) | 1.000 (0.009) | 1.000 (0.000) | 1.000 (0.000) | 0.493 (0.020) | 1.000 (0.000) | 1.000 (0.004) | 0.386 (0.021) | 1.000 (0.004) |
| **IAT10** | 1.000 (0.006) | 1.000 (0.000) | 0.386 (0.024) | 1.000 (0.002) | 1.000 (0.009) | 1.000 (0.012) | 1.000 (0.009) | 1.000 (0.000) | 1.000 (0.000) | 0.659 (0.014) | 0.561 (0.023) | 1.000 (0.000) | 0.542 (0.024) | 1.000 (0.009) | / | 1.000 (0.007) | 1.000 (0.007) | 1.000 (0.000) | 1.000 (0.011) | 0.809 (0.017) | 1.000 (0.006) | 1.000 (0.000) | 0.659 (0.021) | 0.860 (0.012) |
| **IAT11** | 0.386 (0.015) | 1.000 (0.000) | 1.000 (0.000) | 1.000 (0.002) | 1.000 (0.000) | 1.000 (0.006) | 1.000 (0.000) | 1.000 (0.000) | 0.631 (0.020) | 0.758 (0.020) | 0.643 (0.021) | 0.631 (0.018) | 1.000 (0.008) | 0.809 (0.017) | 1.000 (0.007) | / | 1.000 (0.001) | 1.000 (0.002) | 1.000 (0.000) | 1.000 (0.005) | 1.000 (0.003) | 1.000 (0.009) | 1.000 (0.008) | 0.769 (0.020) |
| **IAT12** | 1.000 (0.001) | 1.000 (0.005) | 1.000 (0.002) | 1.000 (0.000) | 1.000 (0.000) | 1.000 (0.006) | 1.000 (0.004) | 0.865 (0.008) | 1.000 (0.008) | 1.000 (0.012) | 1.000 (0.012) | 1.000 (0.003) | 1.000 (0.011) | 1.000 (0.009) | 1.000 (0.007) | 1.000 (0.001) | / | 1.000 (0.007) | 1.000 (0.004) | 1.000 (0.004) | 1.000 (0.012) | 1.000 (0.002) | 1.000 (0.000) | 0.758 (0.023) |
| **IAT13** | 1.000 (0.000) | 1.000 (0.000) | 1.000 (0.005) | 1.000 (0.000) | 1.000 (0.010) | 1.000 (0.000) | 1.000 (0.004) | 1.000 (0.001) | 1.000 (0.000) | 1.000 (0.001) | 0.092 (0.057) | 1.000 (0.000) | 0.963 (0.015) | 1.000 (0.000) | 1.000 (0.000) | 1.000 (0.002) | 1.000 (0.007) | / | 1.000 (0.002) | 0.368 (0.042) | 0.386 (0.033) | 0.731 (0.011) | 0.631 (0.027) | 1.000 (0.011) |
| **IAT14** | 1.000 (0.002) | 1.000 (0.000) | 0.963 (0.012) | 1.000 (0.000) | 1.000 (0.000) | 1.000 (0.002) | 1.000 (0.000) | 1.000 (0.012) | 1.000 (0.004) | 0.960 (0.015) | 0.165 (0.026) | 0.518 (0.025) | 0.514 (0.023) | 1.000 (0.000) | 1.000 (0.011) | 1.000 (0.000) | 1.000 (0.004) | 1.000 (0.002) | / | 0.631 (0.027) | 0.963 (0.019) | 0.758 (0.020) | 1.000 (0.004) | 1.000 (0.010) |
| **IAT15** | 1.000 (0.000) | 1.000 (0.006) | 1.000 (0.001) | 0.814 (0.001) | 1.000 (0.000) | 0.368 (0.013) | 0.514 (0.014) | 1.000 (0.000) | 1.000 (0.008) | 1.000 (0.006) | 0.532 (0.010) | 1.000 (0.000) | 0.814 (0.015) | 0.493 (0.020) | 0.809 (0.017) | 1.000 (0.005) | 1.000 (0.004) | 0.368 (0.042) | 0.631 (0.027) | / | 1.000 (0.001) | 0.860 (0.019) | 1.000 (0.002) | 1.000 (0.015) |
| **IAT16** | 1.000 (0.000) | 1.000 (0.000) | 1.000 (0.000) | 1.000 (0.000) | 0.769 (0.005) | 1.000 (0.000) | 1.000 (0.000) | 1.000 (0.001) | 0.814 (0.018) | 0.454 (0.018) | 0.577 (0.022) | 1.000 (0.008) | 1.000 (0.000) | 1.000 (0.000) | 1.000 (0.006) | 1.000 (0.003) | 1.000 (0.012) | 0.386 (0.033) | 0.963 (0.019) | 1.000 (0.001) | / | 0.865 (0.018) | 1.000 (0.014) | 0.769 (0.022) |
| **IAT17** | 0.914 (0.001) | 1.000 (0.000) | 0.982 (0.005) | 1.000 (0.003) | 1.000 (0.000) | 1.000 (0.000) | 1.000 (0.003) | 0.758 (0.020) | 1.000 (0.000) | 1.000 (0.003) | 1.000 (0.004) | 0.901 (0.016) | 1.000 (0.000) | 1.000 (0.004) | 1.000 (0.000) | 1.000 (0.009) | 1.000 (0.002) | 0.731 (0.011) | 0.758 (0.020) | 0.860 (0.019) | 0.865 (0.018) | / | 1.000 (0.013) | 1.000 (0.005) |
| **IAT18** | 1.000 (0.006) | 1.000 (0.000) | 1.000 (0.005) | 1.000 (0.000) | 1.000 (0.001) | 1.000 (0.000) | 1.000 (0.000) | 1.000 (0.000) | 0.518 (0.015) | 1.000 (0.009) | 1.000 (0.000) | 1.000 (0.008) | 0.769 (0.020) | 0.386 (0.021) | 0.659 (0.021) | 1.000 (0.008) | 1.000 (0.000) | 0.631 (0.027) | 1.000 (0.004) | 1.000 (0.002) | 1.000 (0.014) | 1.000 (0.013) | / | 1.000 (0.011) |
| **IAT20** | 1.000 (0.000) | 1.000 (0.000) | 1.000 (0.006) | 1.000 (0.000) | 0.514 (0.023) | 0.733 (0.014) | 1.000 (0.000) | 1.000 (0.004) | 0.532 (0.017) | 0.769 (0.020) | 1.000 (0.004) | 1.000 (0.000) | 0.276 (0.039) | 1.000 (0.004) | 0.860 (0.012) | 0.769 (0.020) | 0.758 (0.023) | 1.000 (0.011) | 1.000 (0.010) | 1.000 (0.015) | 0.769 (0.022) | 1.000 (0.005) | 1.000 (0.011) | / |

| 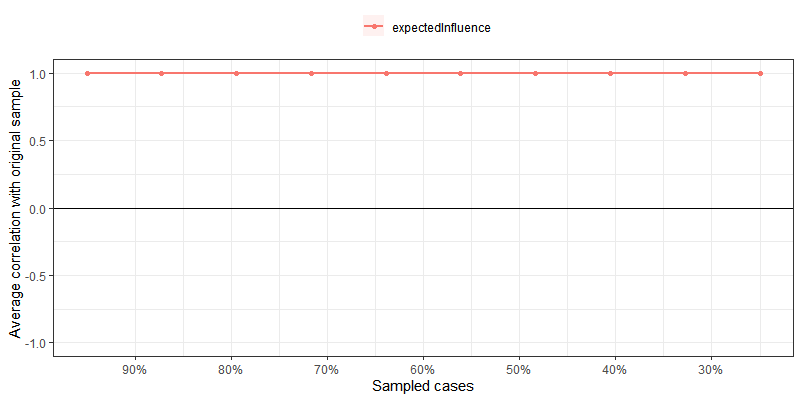 | 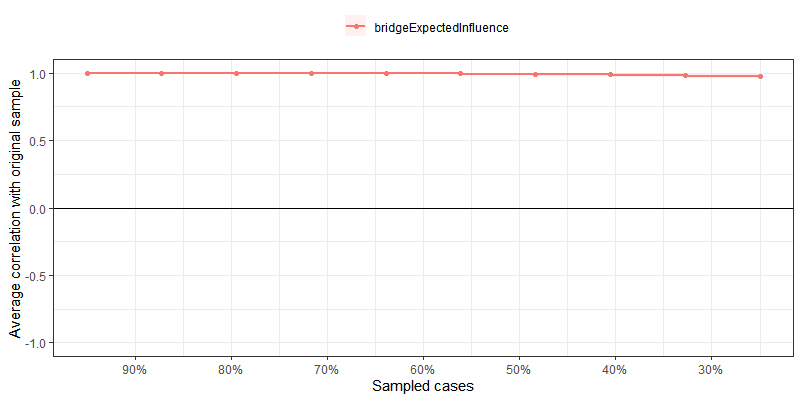 | |  |  |
| --- | --- | --- | --- | --- |
| 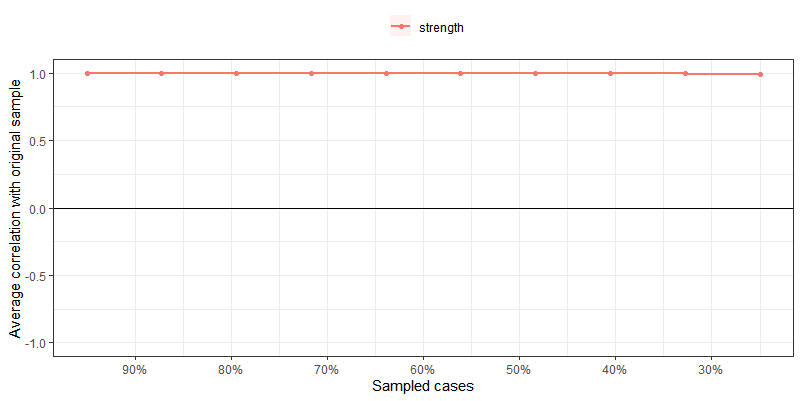 | 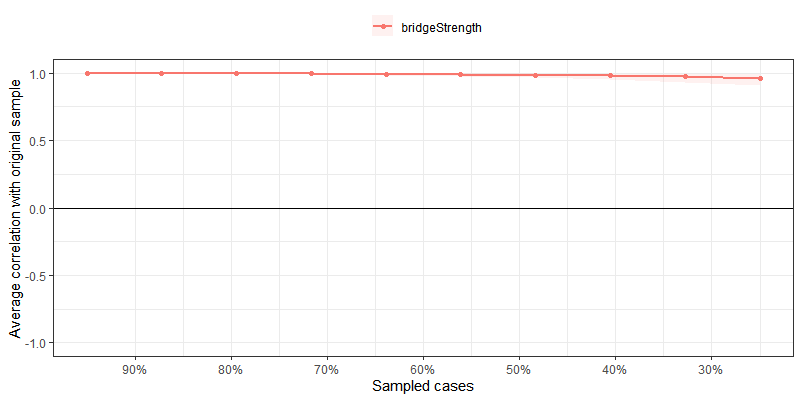 | |  |  |
| 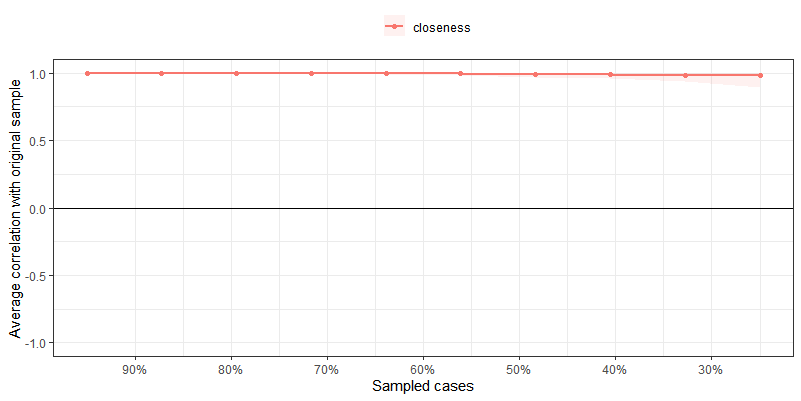 | 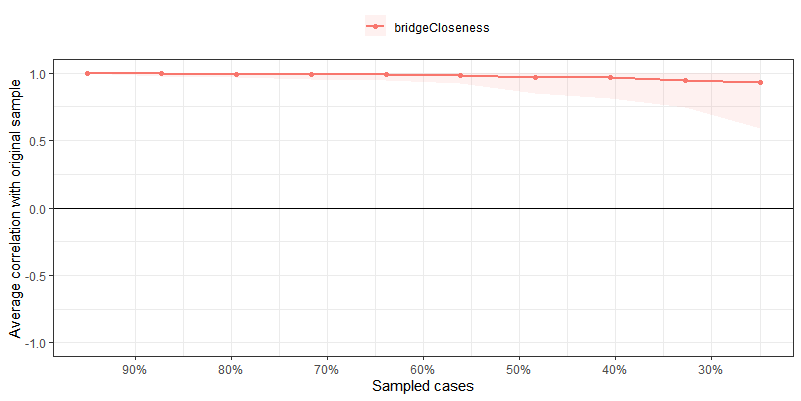 | |  |  |
| 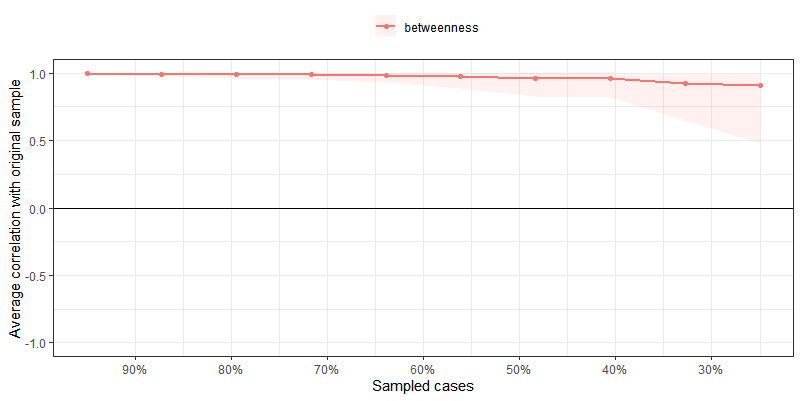 | 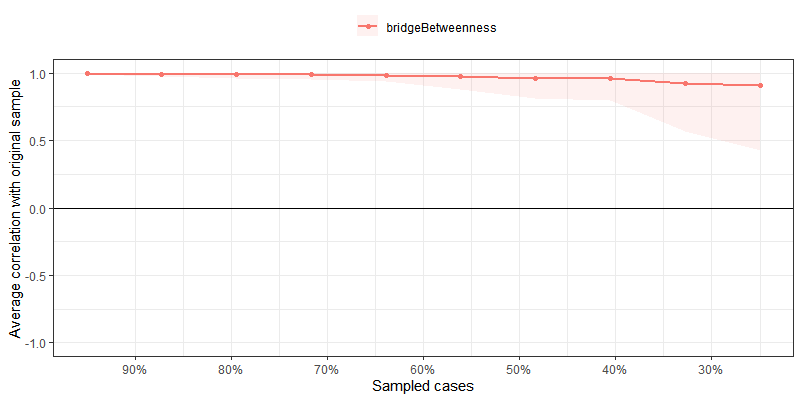 | |  |  |
| 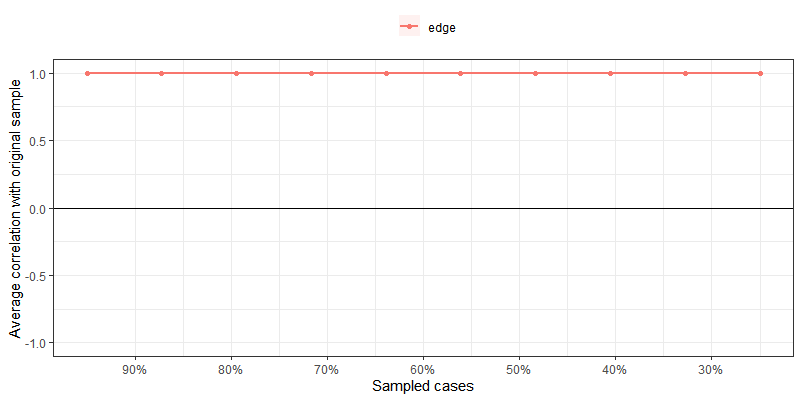 |  | |  |  |
| 1. **Overall populations** | | |  |  |
| 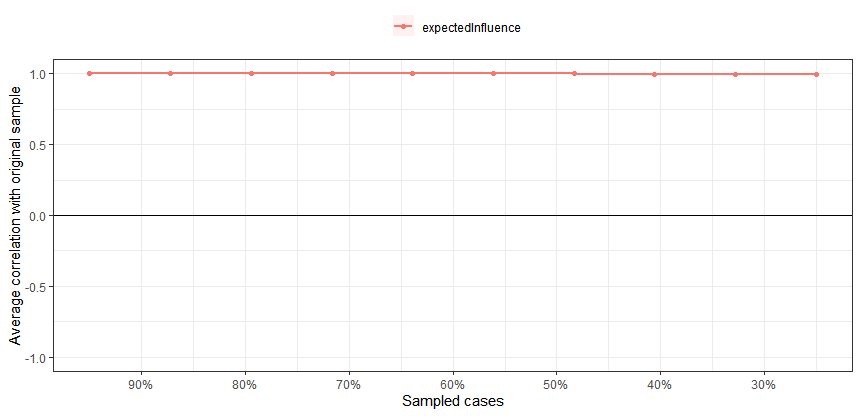 | | | 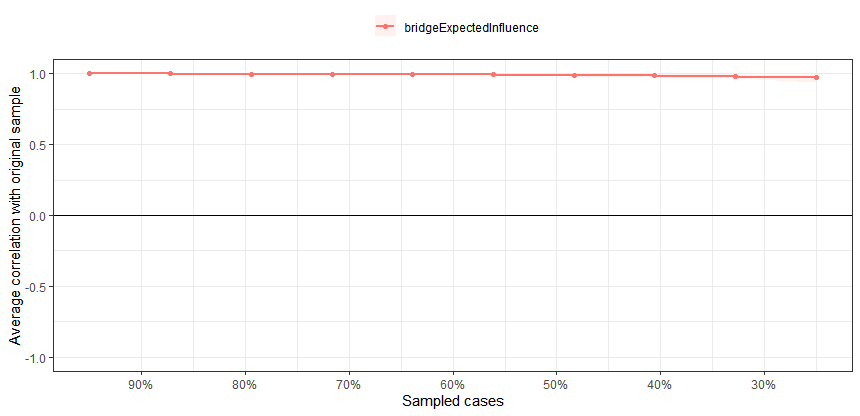 | |
| 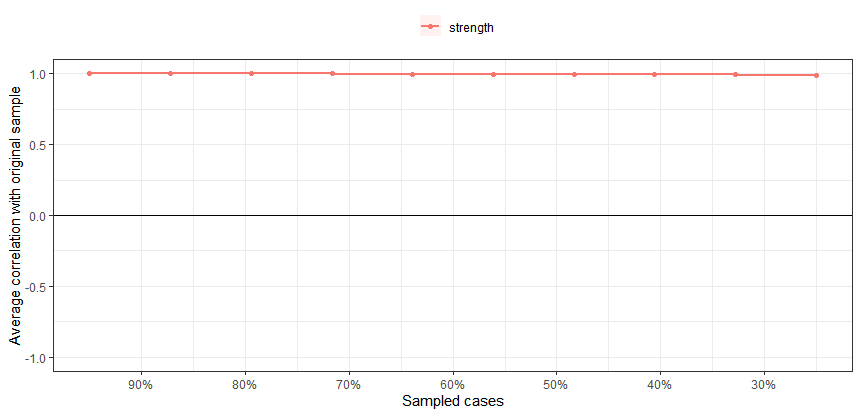 | | | 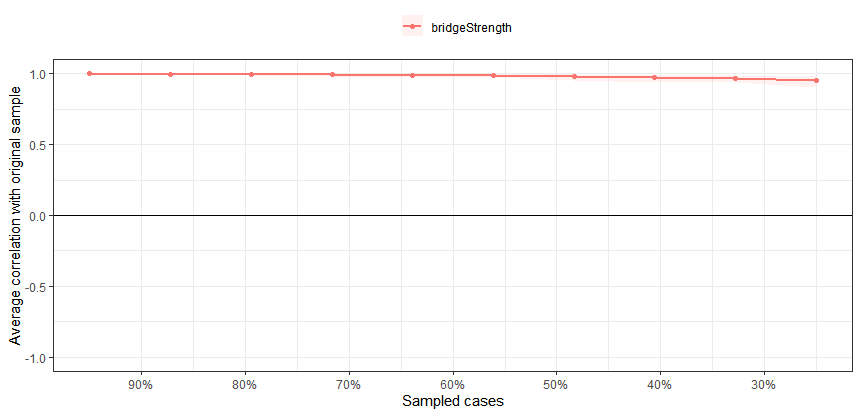 | |
| 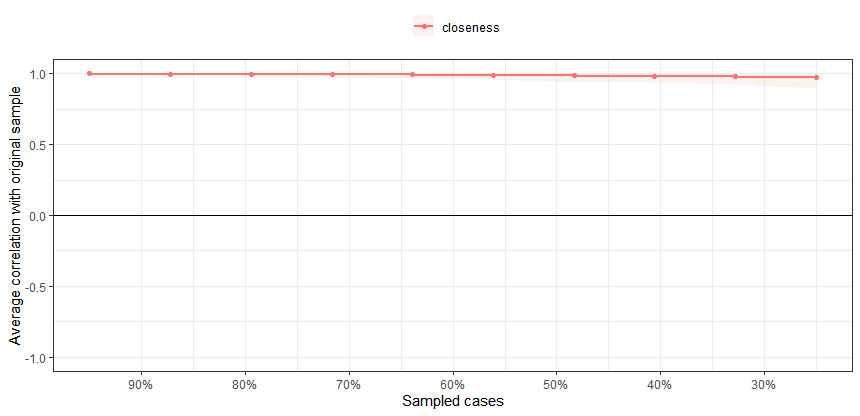 | | | 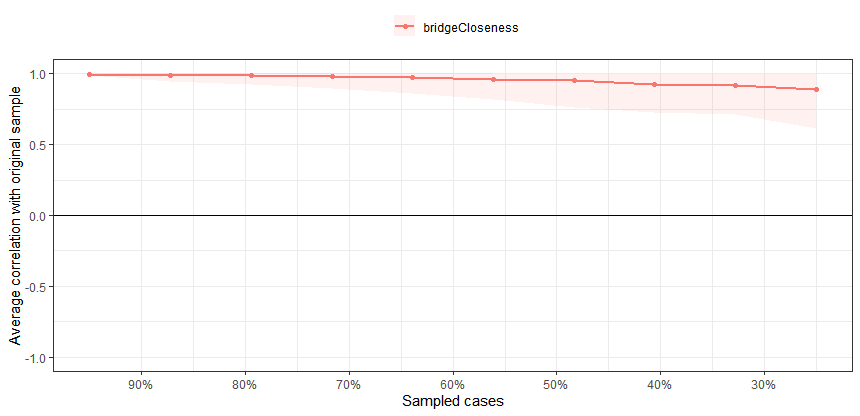 | |
| 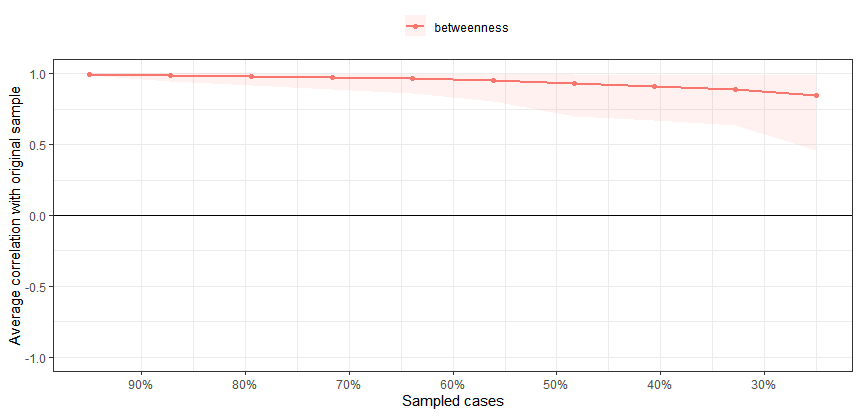 | | | 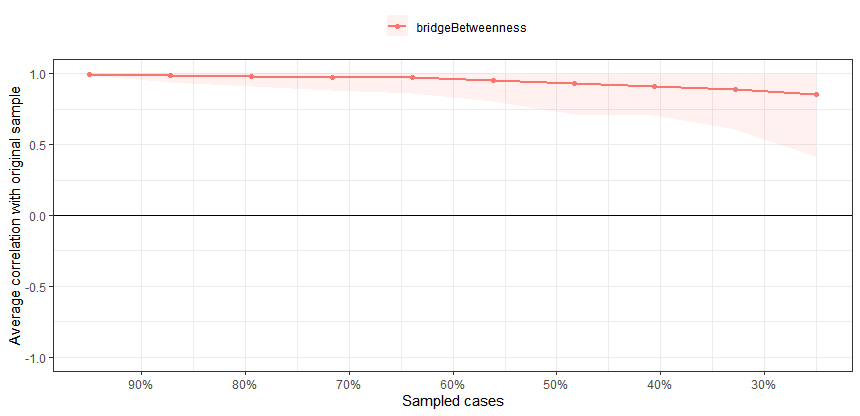 | |
| 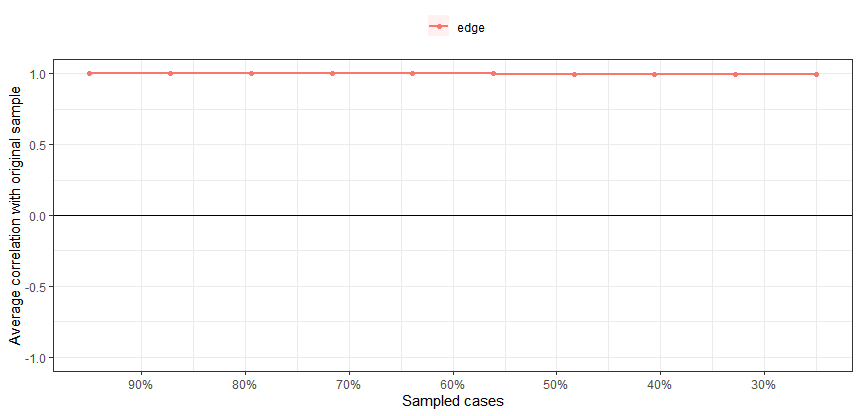 | | |  | |
| 1. **Rural subgroup** | | | | |
| 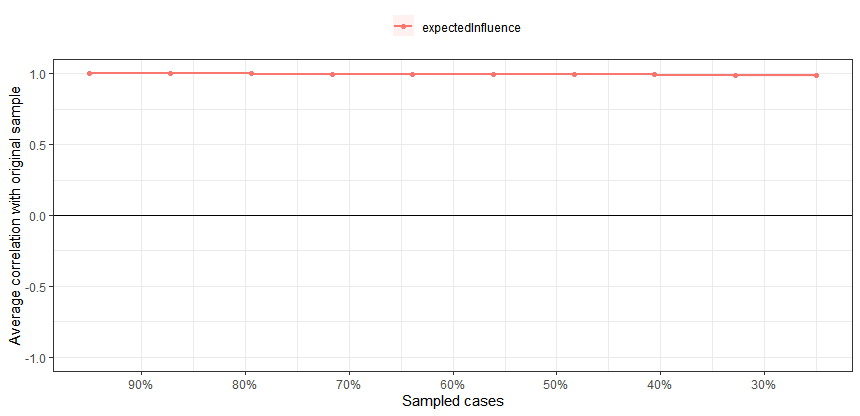 | | | 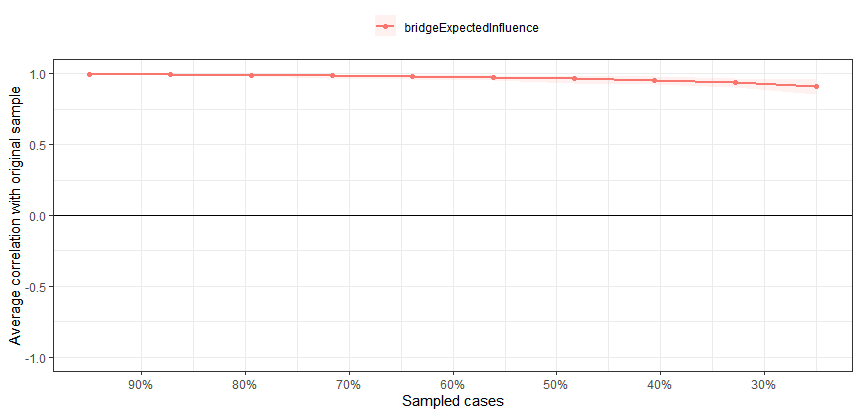 | |
| 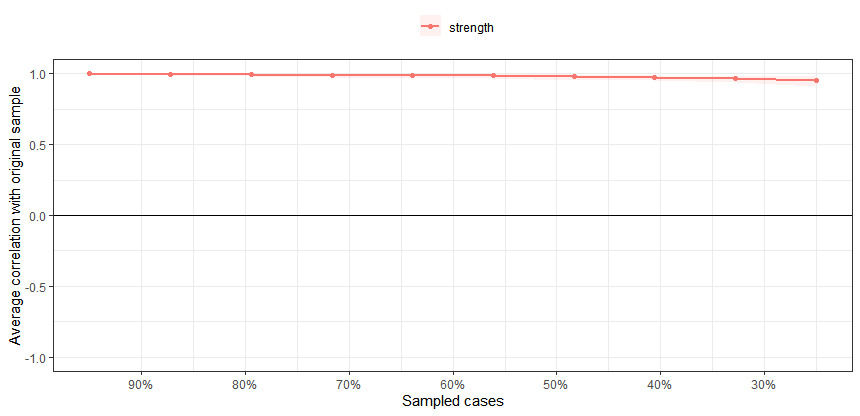 | | | 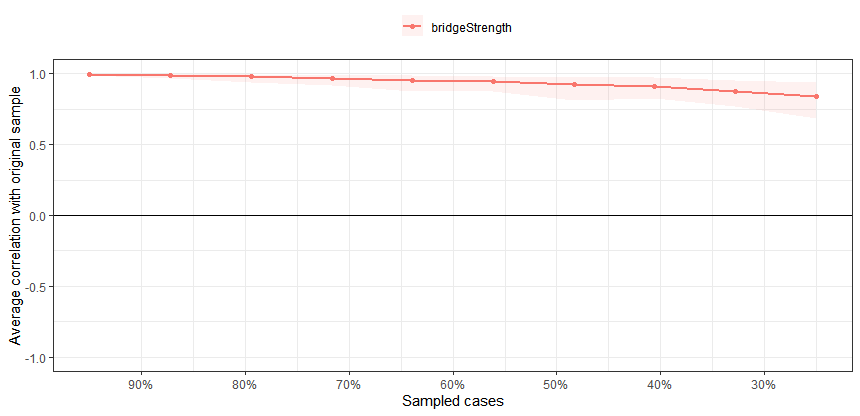 | |
| 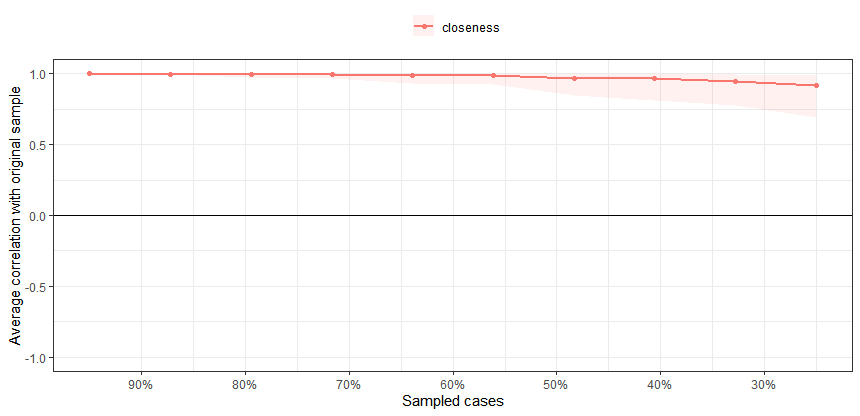 | | | 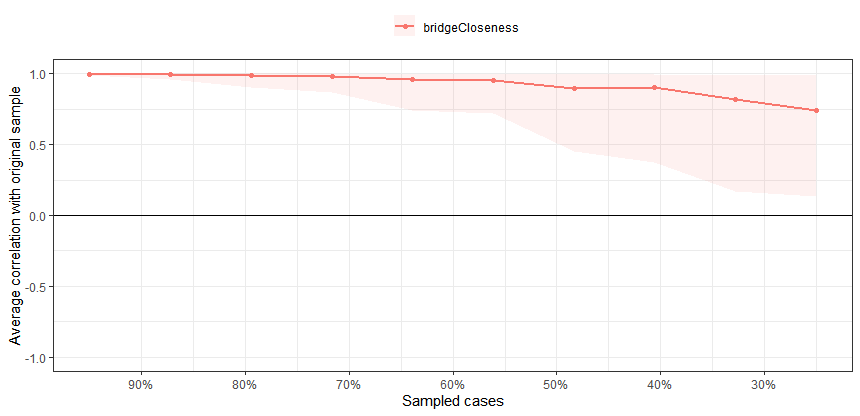 | |
| 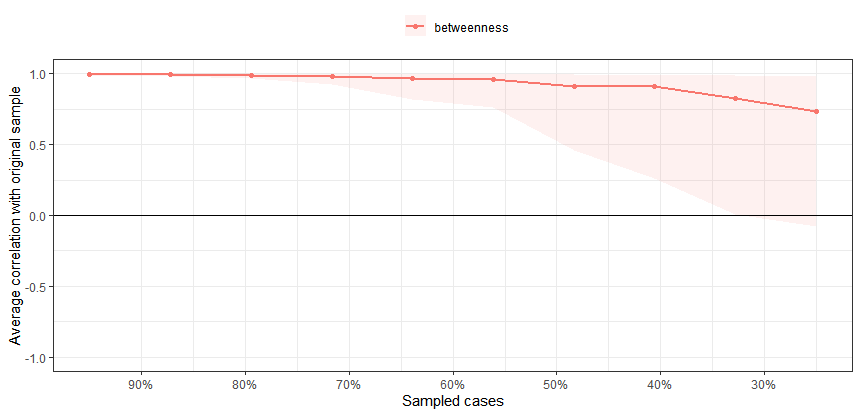 | | | 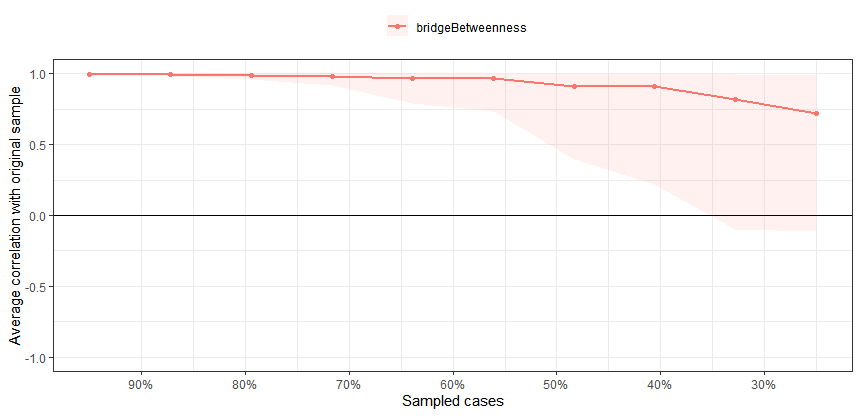 | |
| 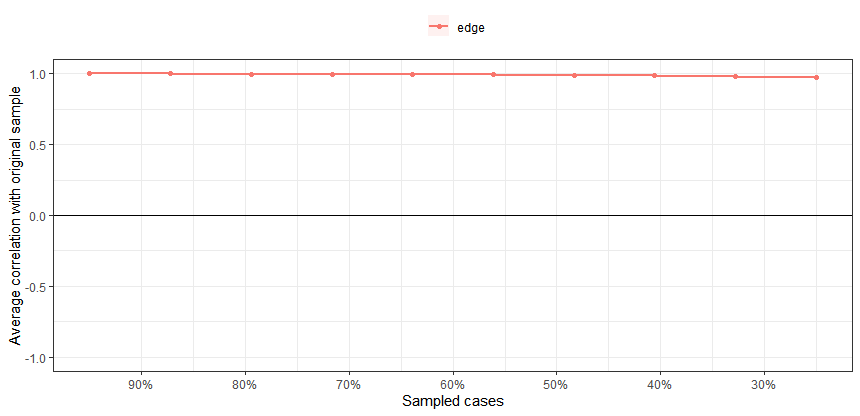 | | |  | |
| 1. **Urban Subgroup** | | | | |
| **Figure S1**  The correlation stability coefficients for stable analysis of the networks while conducting case-dropping bootstrap analysis. | | | | |
| *Note:* A correlation stability coefficient (CS-coefficient) was calculated for evaluating the stability of the networks, showing how much data could be dropped while keeping a correlation of at least 0.70. It requires a CS-coefficient of at least 0.25; results above 0.5 indicate strong network stability. In the figure, red lines represent point estimates, while pale red areas indicate 95% confidence intervals (95% CIs).   1. The CS-coefficients of overall populations are: expected influence: 0.750 (0.672, 1.000), strength: 0.750 (0.672, 1.000), closeness: 0.750 (0.672, 1.000), betweenness: 0.594 (0.517, 0.672), edge weight: 0.750 (0.672, 1.000), bridge expected influence: 0.750 (0.672, 1.000), bridge strength: 0.750 (0.672, 1.000), bridge closeness: 0.750 (0.672, 1.000), bridge betweenness: 0.594 (0.517, 0.672). 2. The CS-coefficients of rural are: expected influence: 0.750 (0.672, 1.000), strength: 0.750 (0.672, 1.000), closeness: 0.750 (0.672, 1.000), betweenness: 0.594 (0.517, 0.672), edge weight: 0.750 (0.672, 1.000), bridge expected influence: 0.750 (0.672, 1.000), bridge strength: 0.750 (0.672, 1.000), bridge closeness: 0.672 (0.594, 0.750), bridge betweenness: 0.594 (0.517, 0.672). 3. The CS-coefficients of urban are: expected influence: 0.750 (0.672, 1.000), strength: 0.750 (0.672, 1.000), closeness: 0.750 (0.672, 1.000), betweenness: 0.517 (0.439, 0.594), edge weight: 0.750 (0.672, 1.000), bridge expected influence: 0.750 (0.672, 1.000), bridge strength: 0.672 (0.594, 0.750), bridge closeness: 0.439 (0.361, 0.517), bridge betweenness: 0.439 (0.361, 0.517). | | | | |

| 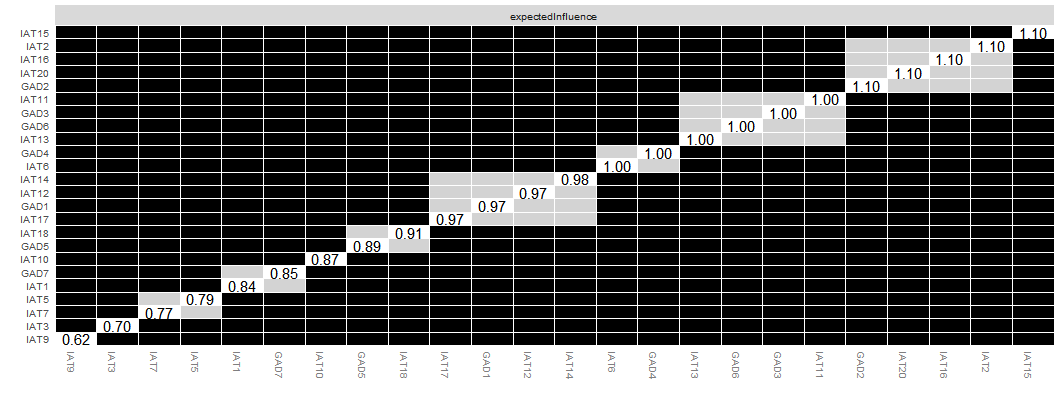 | 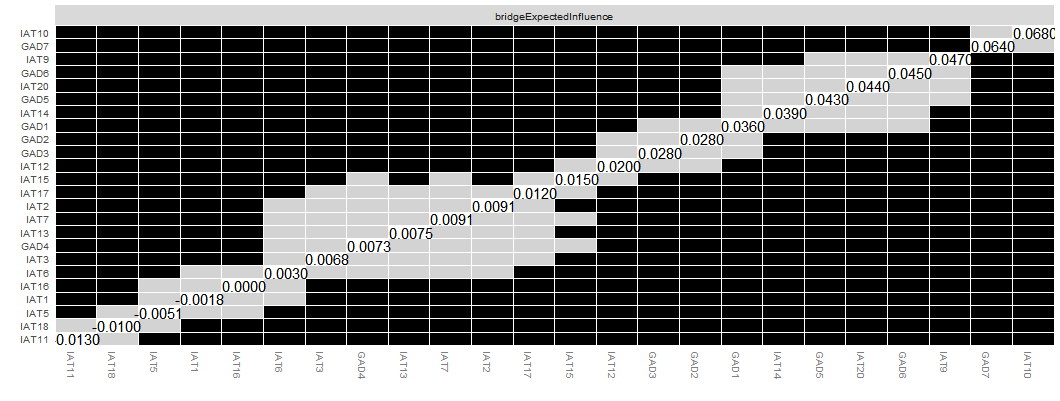 | |
| --- | --- | --- |
| 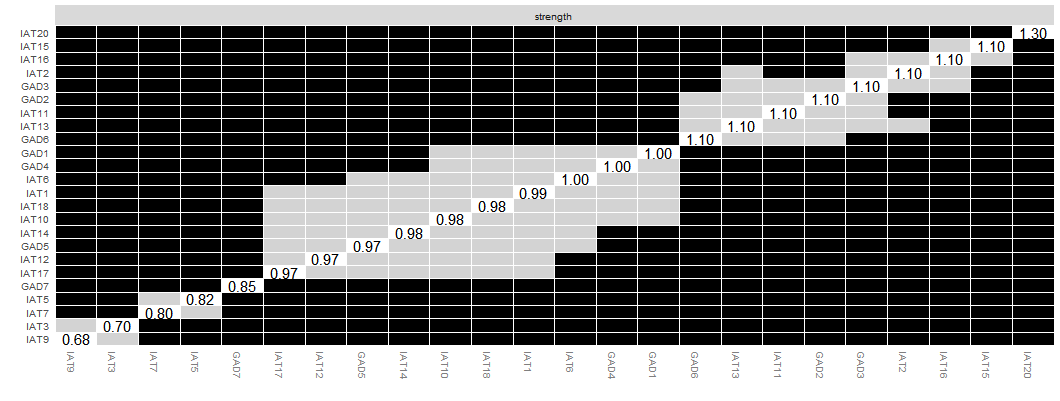 | 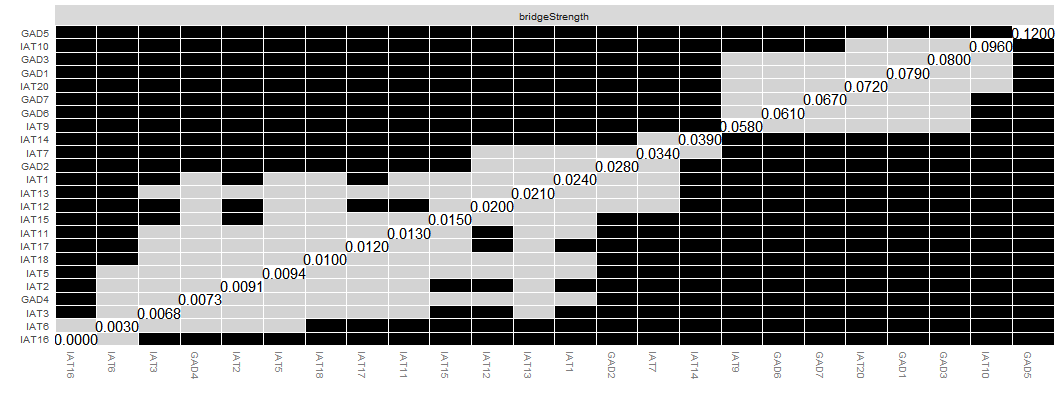 | |
| 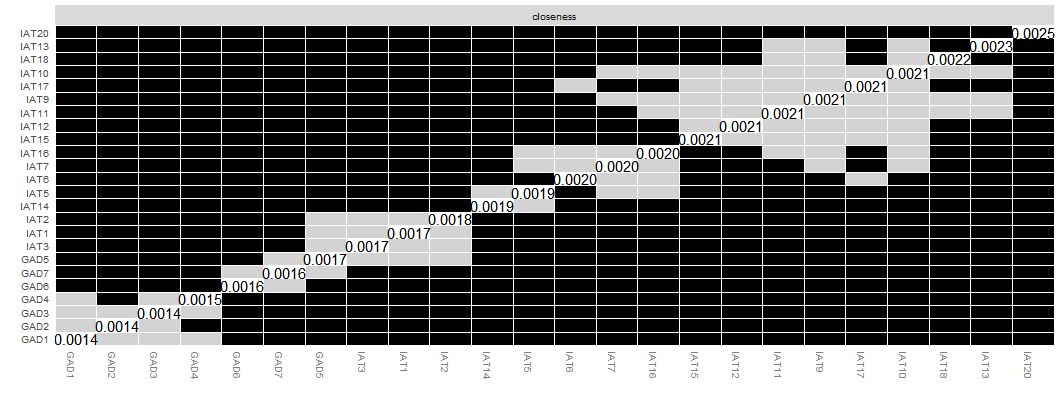 | 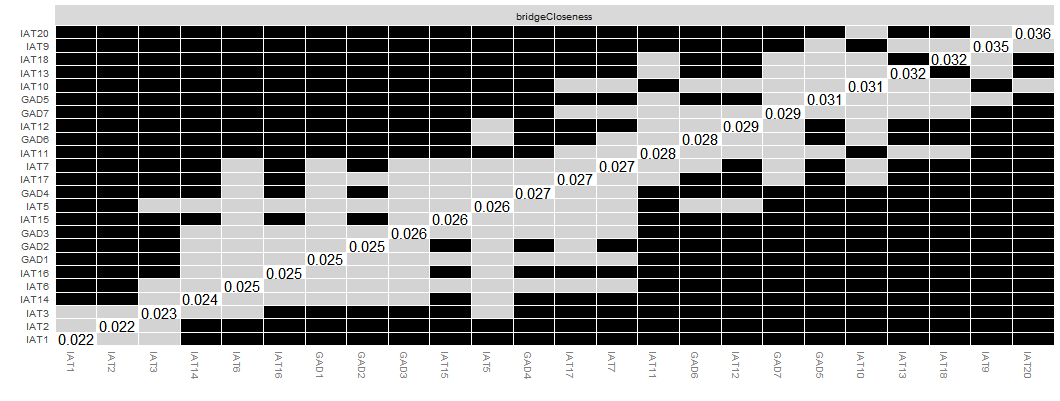 | |
| 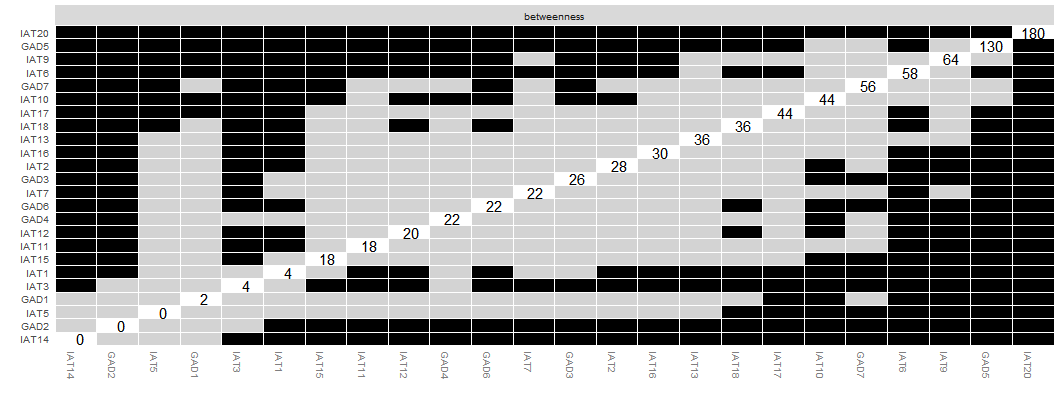 | 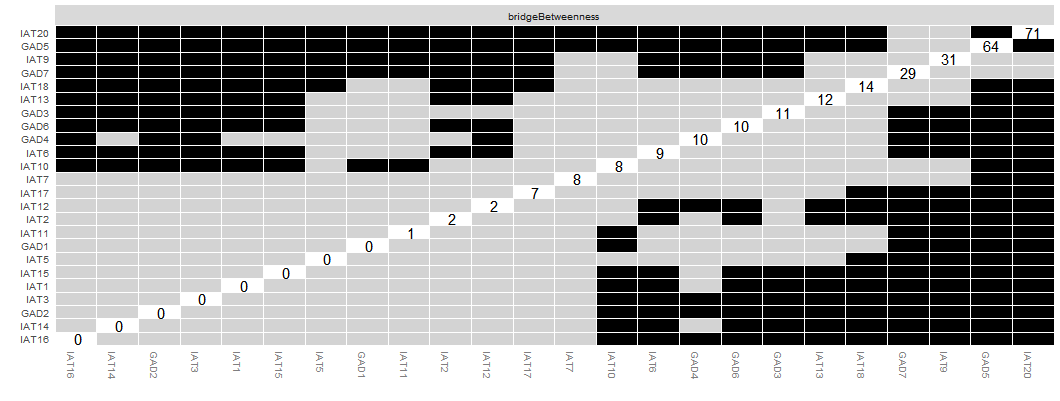 | |
| 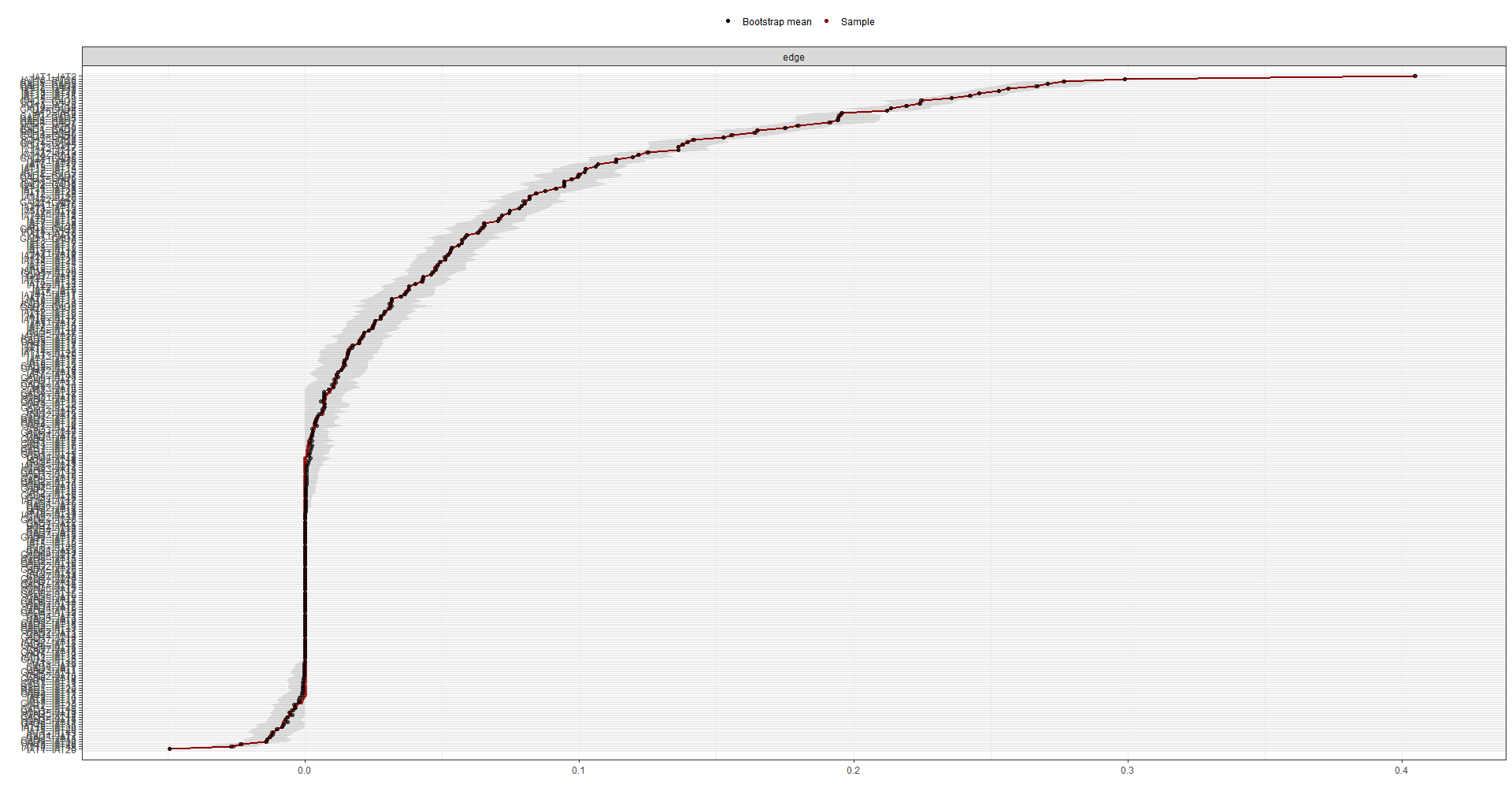   1. **Overall populations** | | |
| 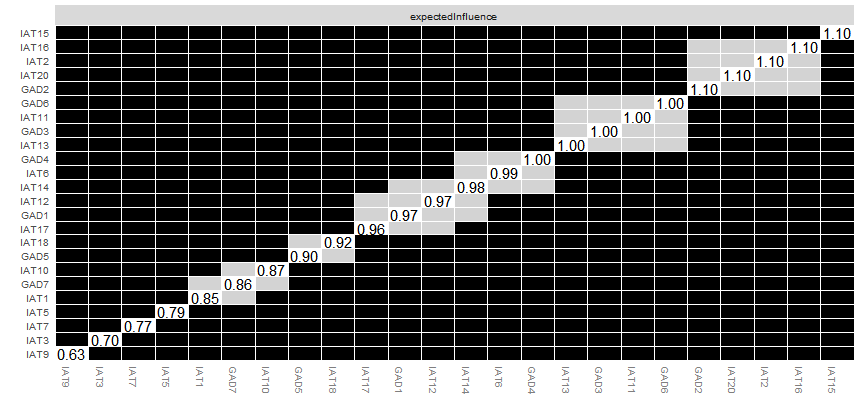 | | 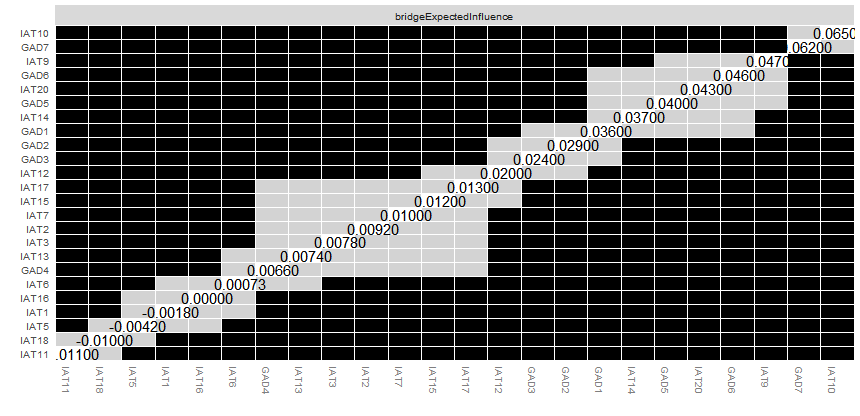 |
| 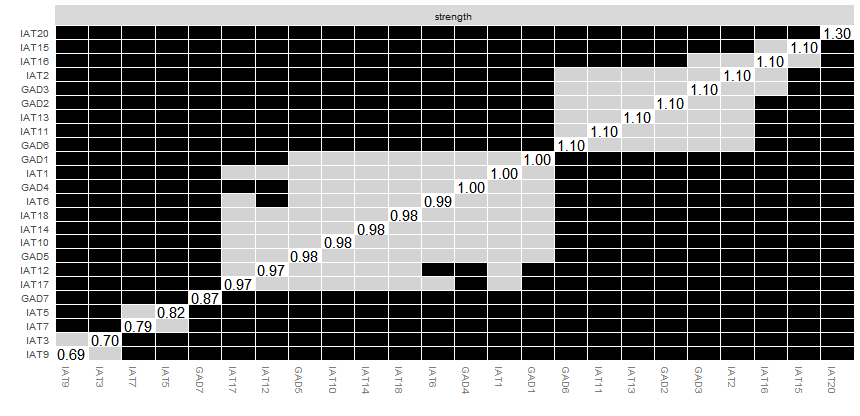 | | 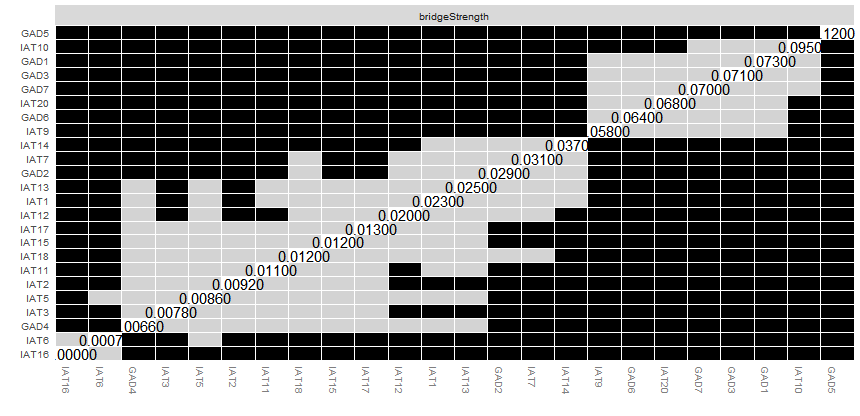 |
| 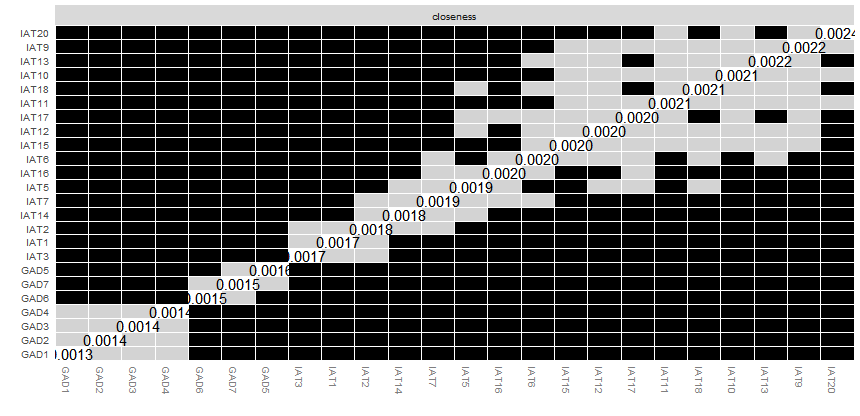 | | 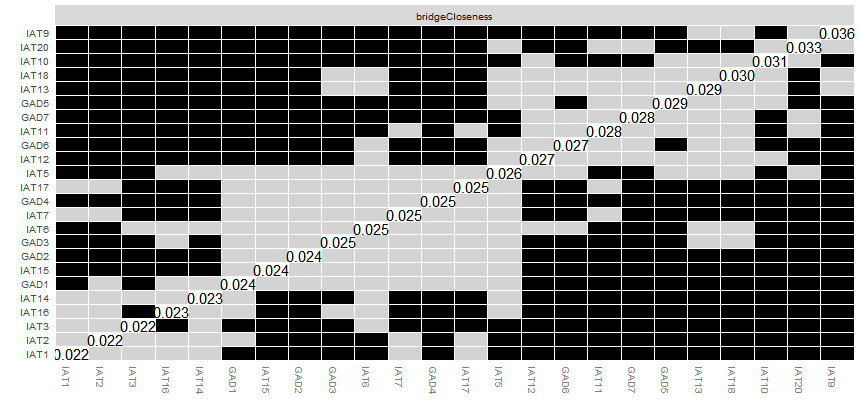 |
| 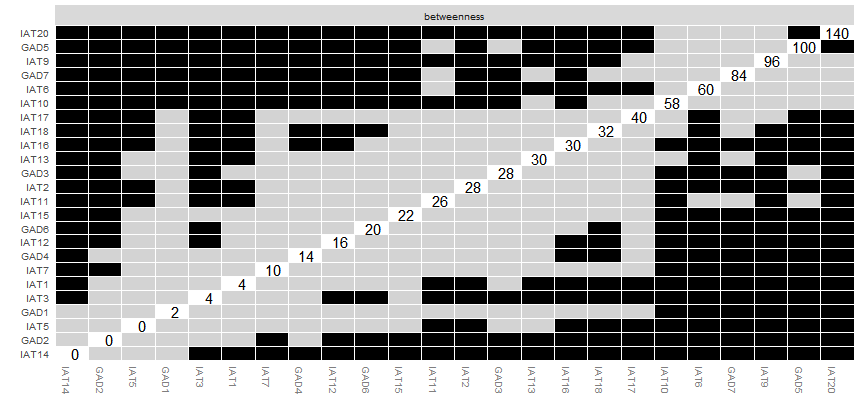 | | 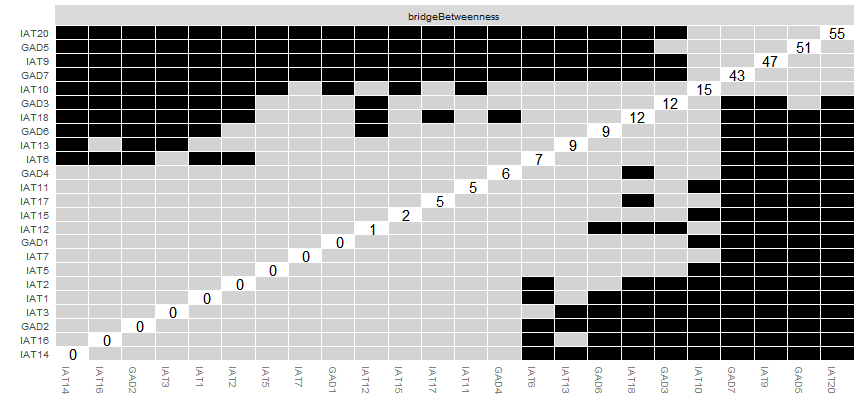 |
| 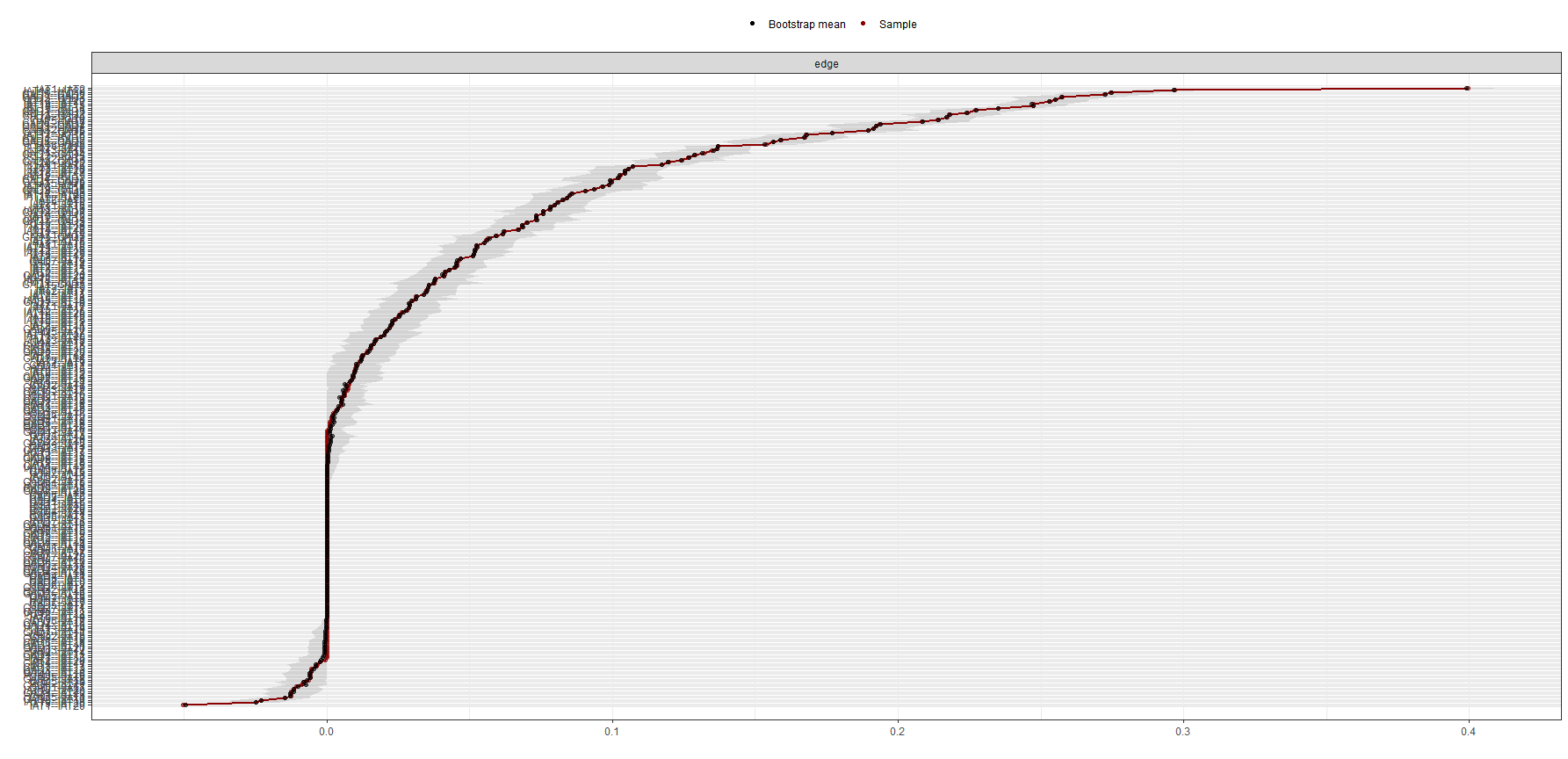   1. **Rural subgroup** | | |
| 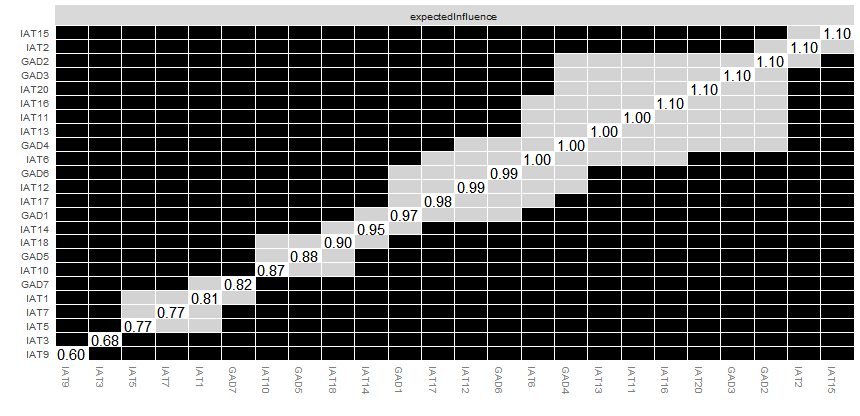 | | 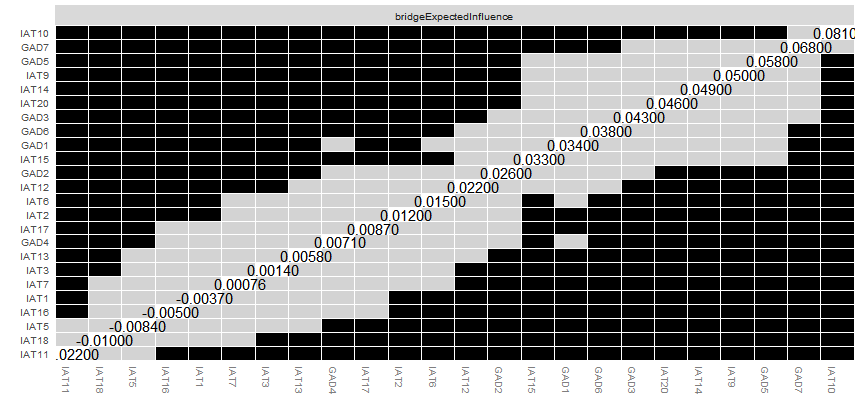 |
| 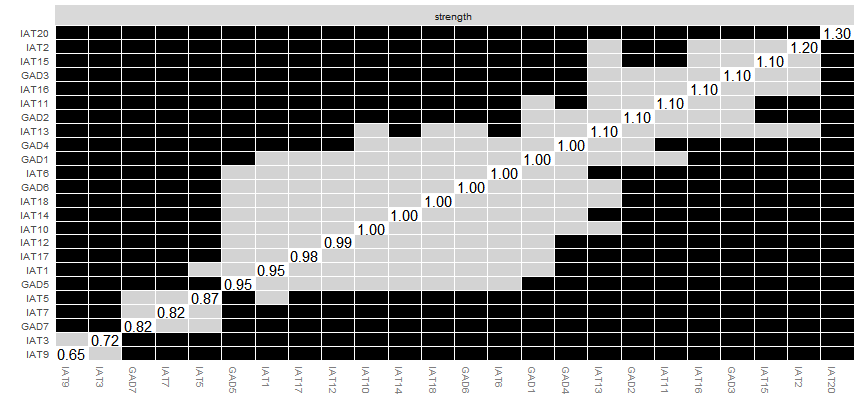 | | 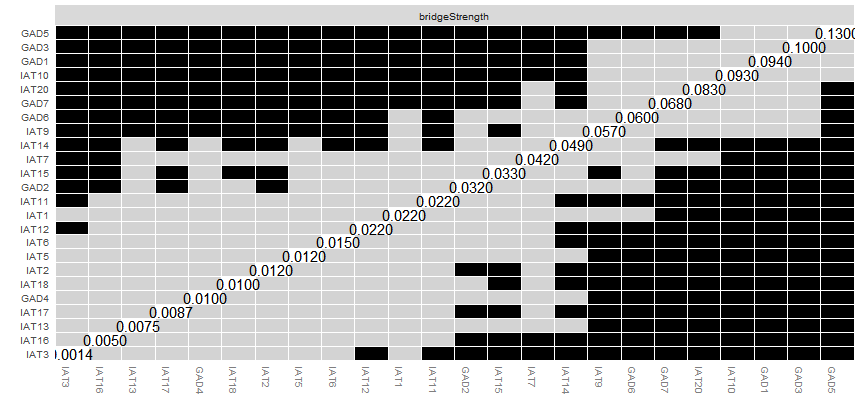 |
| 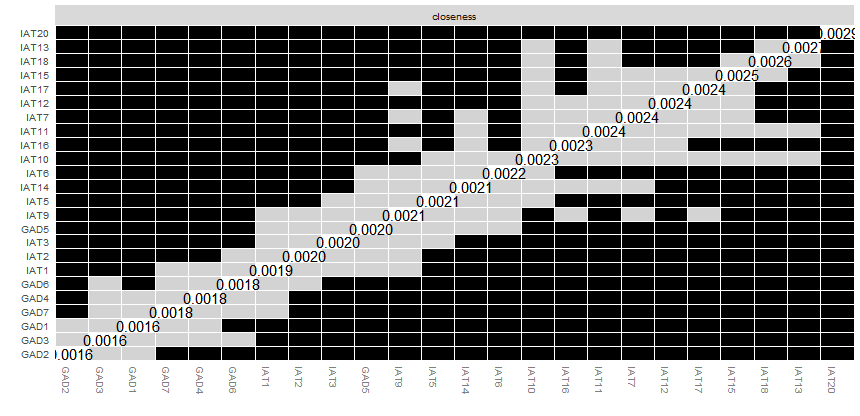 | | 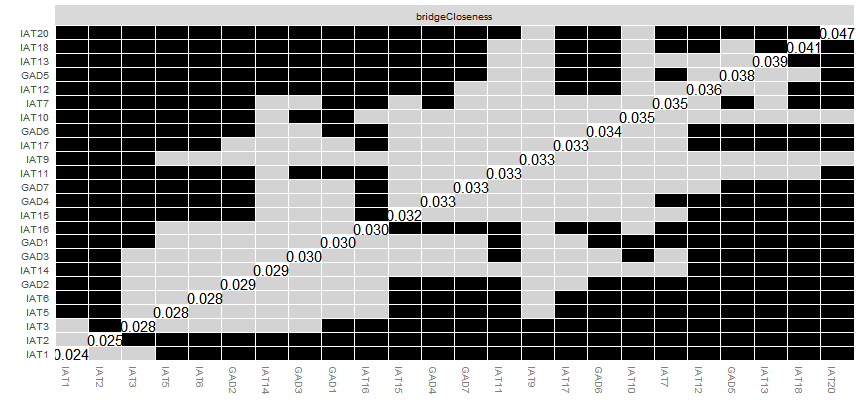 |
| 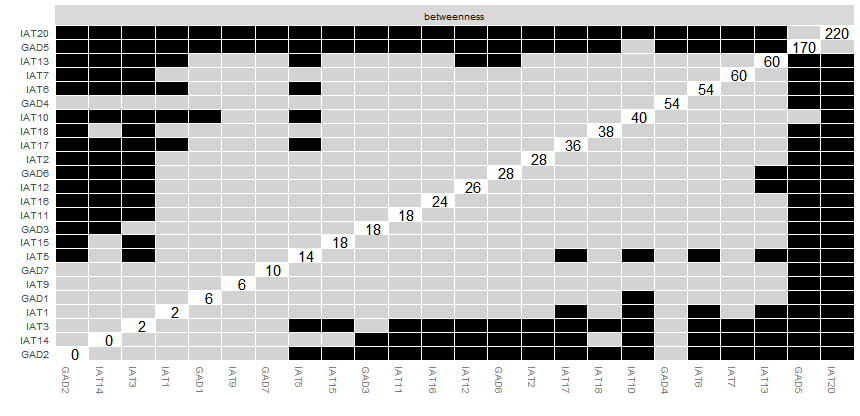 | | 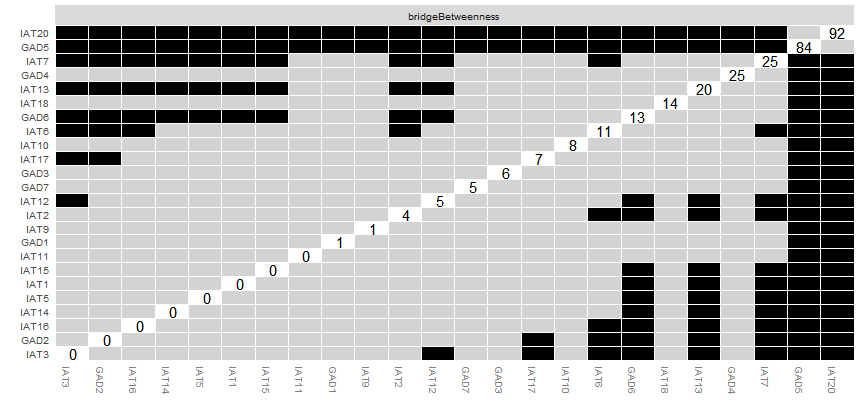 |
| 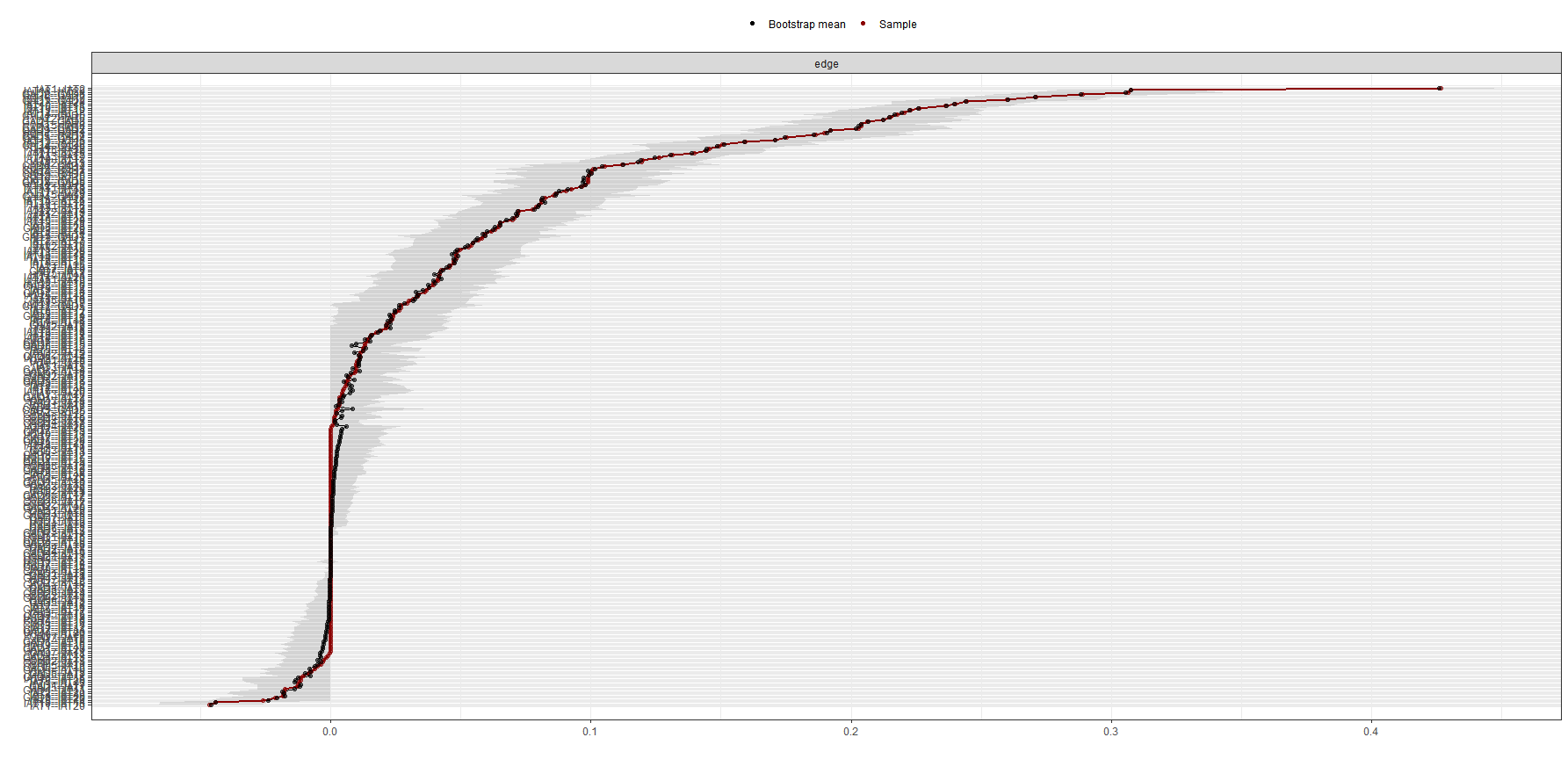   1. **Urban subgroup** | | |
| **Figure S2**  The analysis of *95% CIs* overlap for stable analysis of the networks while conducting case-dropping bootstrap analysis. | | |
| *Note:* For assessing conventional network model reliability and stability, 1,000-case-dropping bootstrap simulation was conducted to estimate whether there are overlaps of *95% CIs* for the indices of two symptoms. Non-overlapping *95% CIs* indicated significant differences, while overlap means no significance. Gray squares indicate overlapping *95% CIs* for the indices of two symptoms and no significant differences, and black squares indicate no overlap and mean significance. For edge weights, the red dots represent original data, the black dots represent point estimates through 1,000 case-dropping bootstrap analysis, and the gray areas indicate the *95% CIs*. | | |

| **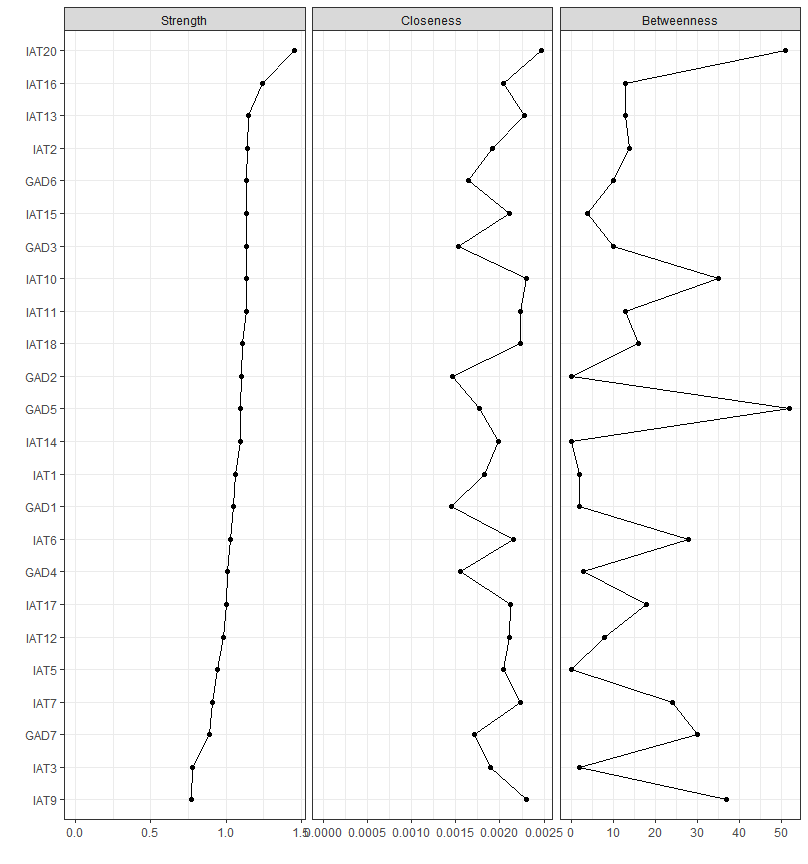** | **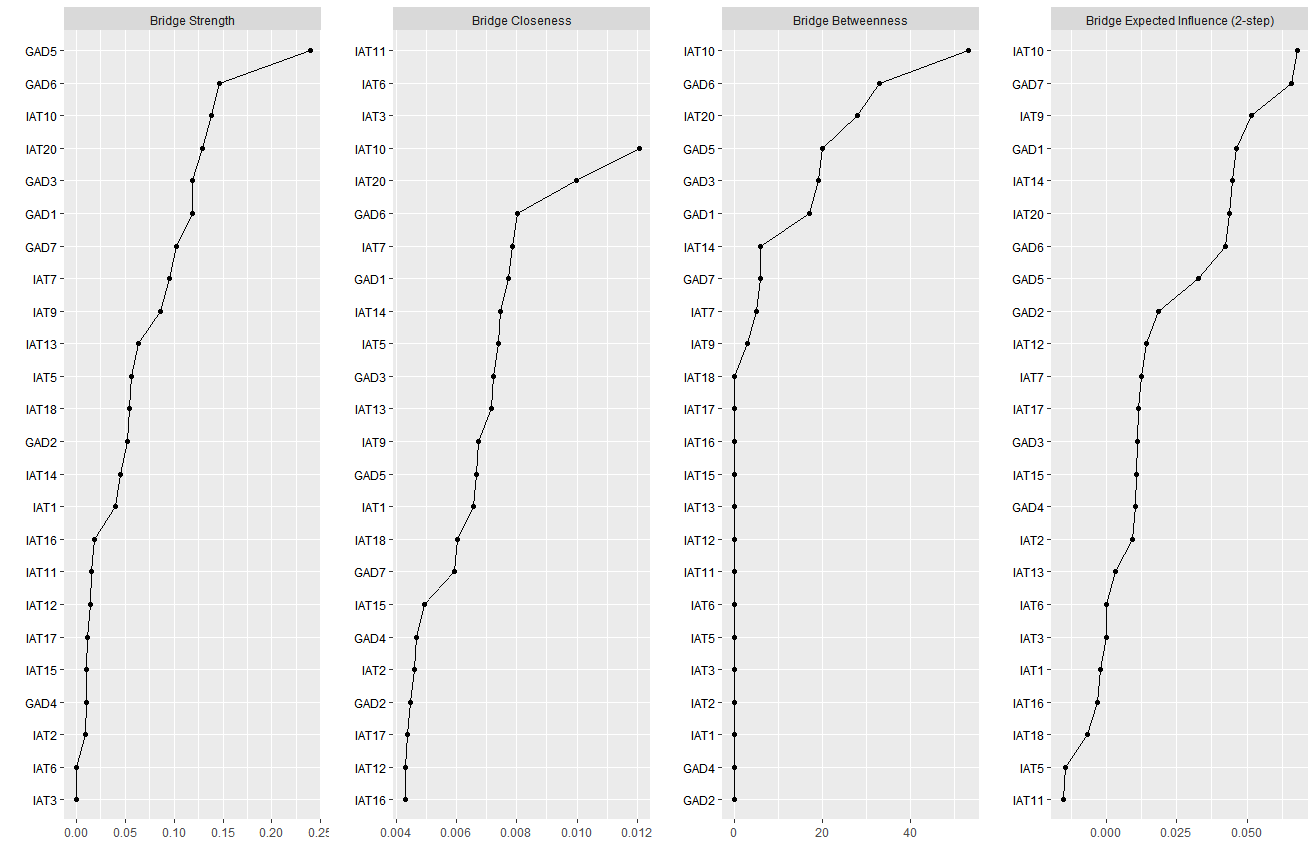** |
| --- | --- |
| 1. **Overall populations** | |
| **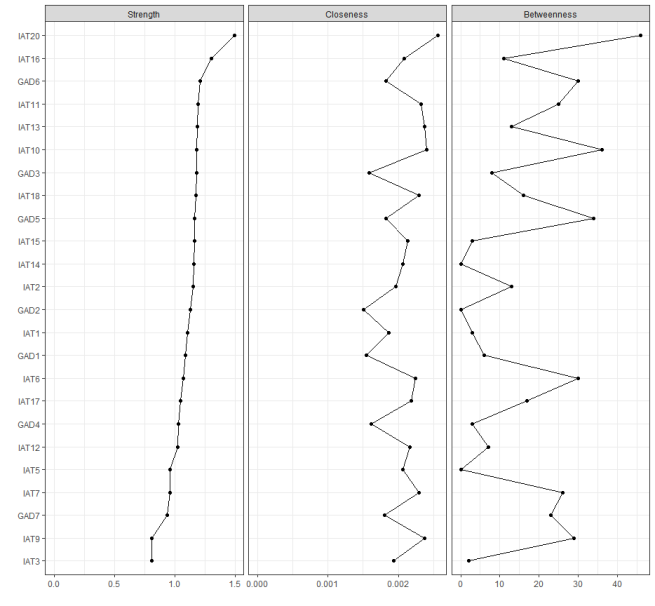** | **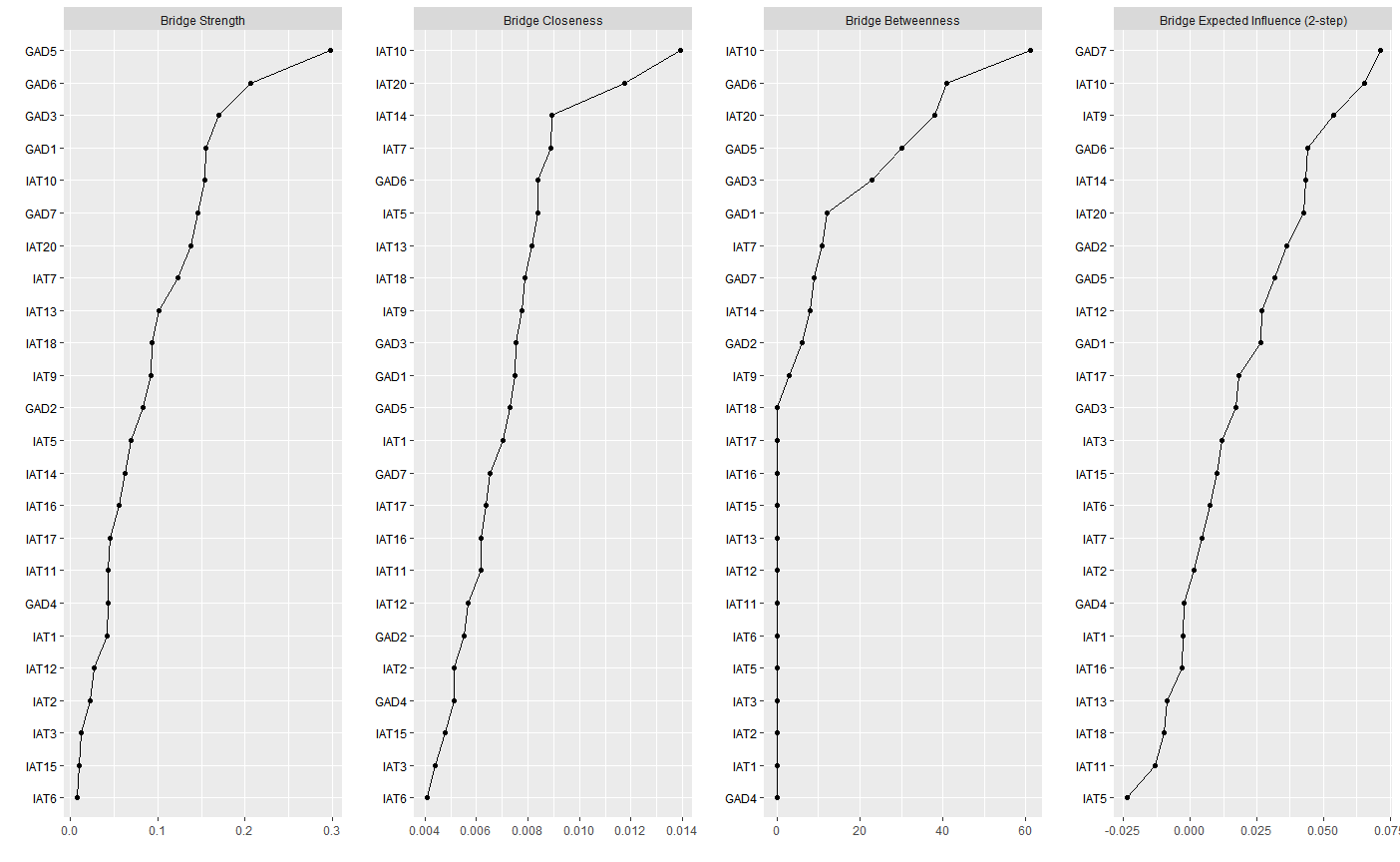** |
| 1. **Rural subgroup** | |
| **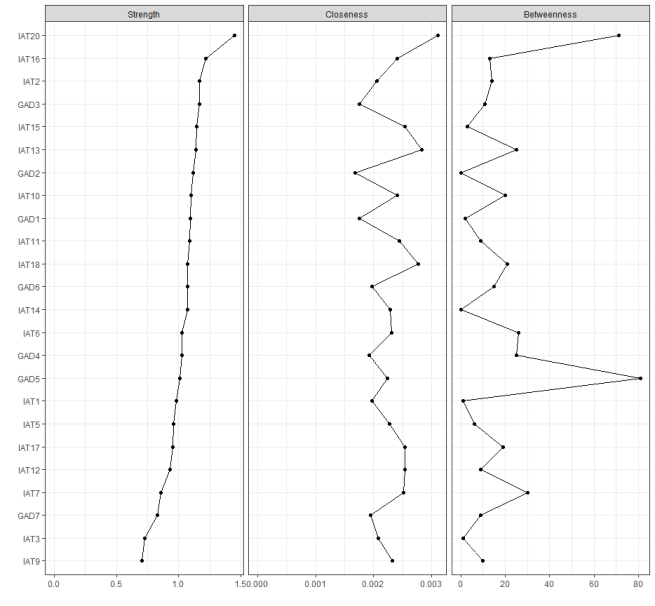** | **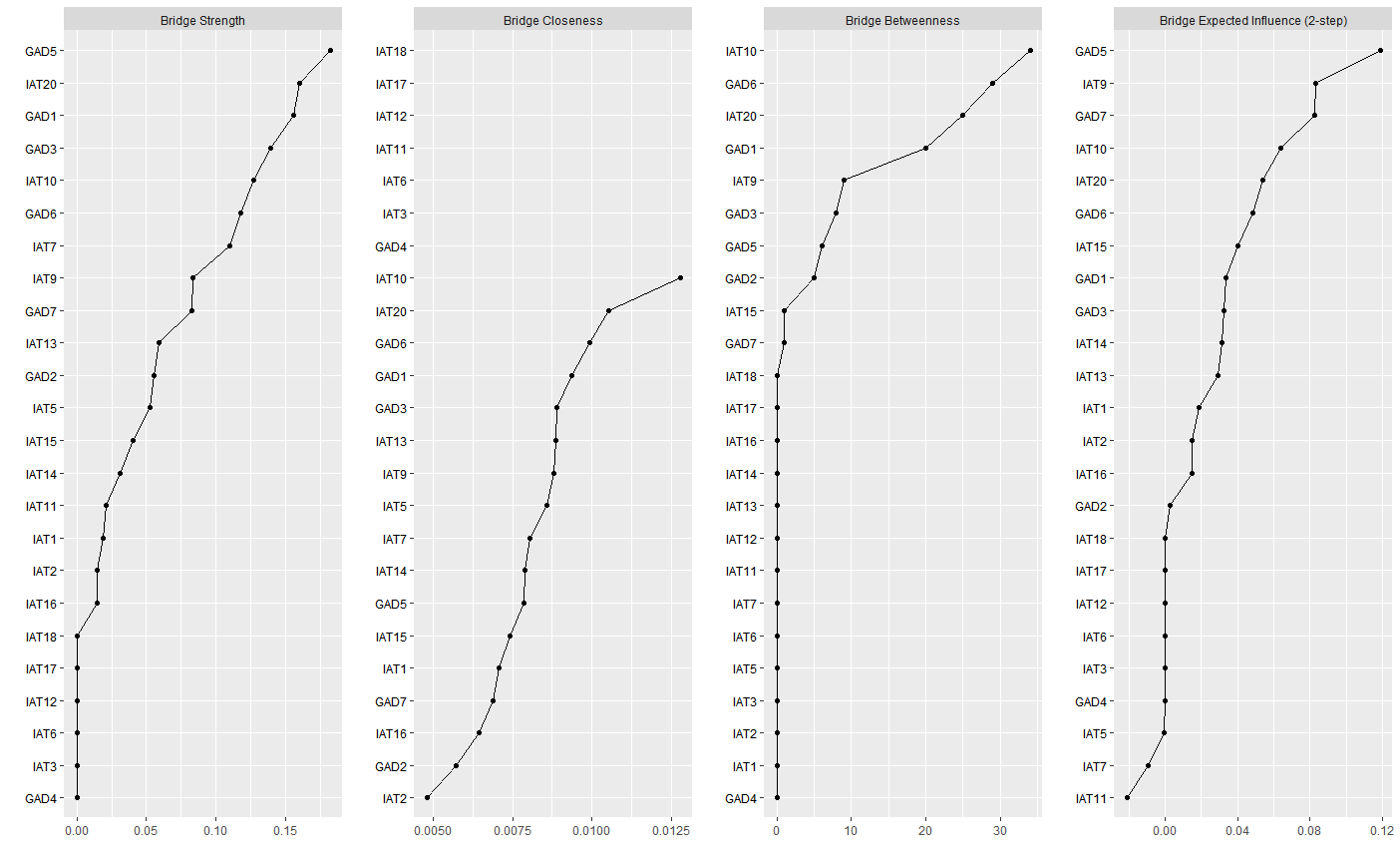** |
| 1. **Urban subgroup** | |
| **Figure S3**  Other supplementary influential indices of symptoms in the anxiety and IA combined network. | |
